# Exploring the Neuroprotective Effects of Walnut (*Juglans regia* L.) Against Cognitive Impairment in Alzheimer’s Disease Through the MAPK-mTORC1-TFEB-Mediated Endolysosomal Pathway: A Network Pharmacology and Molecular Docking Approach

**DOI:** 10.3390/ijms27188288

**Published:** 2026-09-17

**Authors:** Chih-Ting Lin, Kuan-Tso Chen, Yu-Chiang Hung

**Affiliations:** 1 Department of Chinese Medicine, E-Da Cancer Hospital, Kaohsiung 82445, Taiwan; gril20708@gmail.com (C.-T.L.); sugarplant51@hotmail.com (K.-T.C.); 2 Institute of Traditional Medicine, National Yang Ming Chiao Tung University, Taipei 11221, Taiwan; 3 School of Chinese Medicine for Post-Baccalaureate, I-Shou University, Kaohsiung 84001, Taiwan; 4 Department of Chinese Medicine, Institute of Traditional Medicine, National Yang Ming Chiao Tung University, Taipei 11221, Taiwan; 5 Linsen Chinese Medicine and Kunming Branch, Taipei City Hospital, Taipei 10450, Taiwan

**Keywords:** walnut (*Juglans regia* L.), Alzheimer’s disease, cognitive impairment, MAPK-mTORC1-TFEB axis, endolysosomal homeostasis

## Abstract

Alzheimer’s disease (AD) is a complex neurodegenerative disorder with multifaceted pathogenesis. Given the clinical need for multi-target interventions, this study elucidated the neuroprotective mechanisms of walnut (*Juglans regia* L.) against AD-related cognitive impairment. Methods: We characterized 25 bioactive constituents (fatty acids and ellagitannins) and constructed a protein–protein interaction (PPI) network. GO/KEGG enrichment, integrated with Ingenuity Pathway Analysis (IPA), deciphered pharmacological pathways, followed by molecular docking to evaluate ligand–target binding thermodynamics. Network analysis revealed that walnut phytochemicals synergistically modulate the MAPK-mTORC1-TFEB-mediated endolysosomal homeostasis pathway. Molecular docking indicated that ellagitannins (strictinin, pedunculagin, tellimagrandin I) exhibit robust affinities for upstream RAS/RAF/MEK kinases, suppressing pro-pathogenic signaling. Concurrently, gallic acid targeted ABCA1 and TFEB, promoting TFEB activation and nuclear translocation to enhance beta-amyloid clearance and lysosomal biogenesis. This study provides evidence that walnut attenuates AD pathology through “multi-component” reinforcement of the endolysosomal defense system, establishing a mechanistic foundation for walnut as a promising functional food.

## 1. Introduction

Alzheimer’s disease (AD) is a devastating and progressive neurodegenerative disorder that poses an unprecedented global health challenge, currently affecting over 55 million individuals worldwide—a figure projected to nearly triple to 139 million by 2050 [1,2]. The pathological hallmarks of AD, primarily involving the extracellular accumulation of Aβ plaques and the intracellular formation of neurofibrillary tangles, are driven by a highly interconnected network of disrupted signaling cascades, including rampant neuroinflammation, metabolic dysfunction, and severe impairments in autophagic–lysosomal clearance [3]. Despite decades of intensive research, the therapeutic landscape remains highly constrained. While recently developed Aβ-directed monoclonal antibodies, such as Lecanemab, revealed a 27% slowing of clinical decline in the Clarity AD trial (representing a 1.21-point difference on the Clinical Dementia Rating Sum of Boxes scale over 18 months), these benefits are often offset by significant safety concerns. Specifically, approximately 21.3% of treated patients encounter amyloid-related imaging abnormalities, such as brain edema or microhemorrhages [4,5,6]. This high attrition rate in conventional “one-drug–one-target” paradigms suggests that AD pathogenesis is far more complex than a single isolated pathway, highlighting an urgent need for multi-target therapeutic strategies capable of simultaneously modulating these redundant and interconnected pathogenic networks [7,8].

From the perspective of Traditional Chinese Medicine (TCM), cognitive decline in AD is deeply linked to the depletion of “Kidney Essence” and the subsequent “Emptiness of the Marrow Sea,” suggesting that neuroprotection can be achieved by reinforcing the kidney–brain axis to restore cognitive vitality. Walnut (WN), *Juglans regia* L., revered in TCM for its ability to “tonify the Kidney and nourish the Brain,” is rich in bioactive compounds such as large hydrolyzable ellagitannins (e.g., strictinin, pedunculagin, and tellimagrandin I) and small phenolic acids like gallic acid [9,10,11,12]. Epidemiological studies, including data from the PREvención con DIeta MEDiterránea and National Health and Nutrition Examination Survey, indicate that regular walnut consumption is associated with a 30% to 50% reduction in the risk of age-related cognitive decline and a 13% to 20% lower prevalence of cognitive impairment in older adults, with improvements observed across cognitive domains such as story recall, Digit Symbol Substitution, and reaction time [13,14,15]. In vivo studies in transgenic AD mouse models further reveal that walnut-enriched diets can reduce Aβ plaque burden by 30–40%, lower lipid peroxidation markers, and enhance antioxidant enzymes like superoxide dismutase [16]. Despite these promising findings, the specific molecular targets and systemic mechanisms through which walnut constituents facilitate the clearance of toxic protein aggregates—particularly within the endolysosomal system—remain underexplored [17]. Natural products like walnuts have shown remarkable multi-target efficacy and safety, and while their antioxidant properties are well-documented, further research is needed to fully understand their role in bridging nutritional intervention with the molecular restoration of lysosomal homeostasis [18].

In this study, we employed an integrative network pharmacology and computational biology approach—incorporating C-T/PPI network modeling, GO/KEGG enrichment, IPA signaling analysis, and molecular docking simulations—to decipher the neuroprotective mechanisms of 25 core walnut metabolites against AD [7,8]. We hypothesize that walnut phytochemicals exert neuroprotection through a synergistic dual-modulation strategy: inhibiting pro-pathogenic MAPK kinase signaling while simultaneously activating the TFEB-mediated CLEAR gene network to restore lysosomal homeostasis. By delineating this complex pharmacological landscape, this research aims to establish a robust mechanistic foundation for walnut as an evidence-based functional food intervention, offering innovative avenues for future drug–food integration strategies in the management of neurodegenerative diseases.

## 2. Results

### 2.1. Identification of Bioactive Compounds and Overlapping Target Genes of Juglans regia L. in Alzheimer’s Disease and Cognitive Impairment

**Figure 1:**
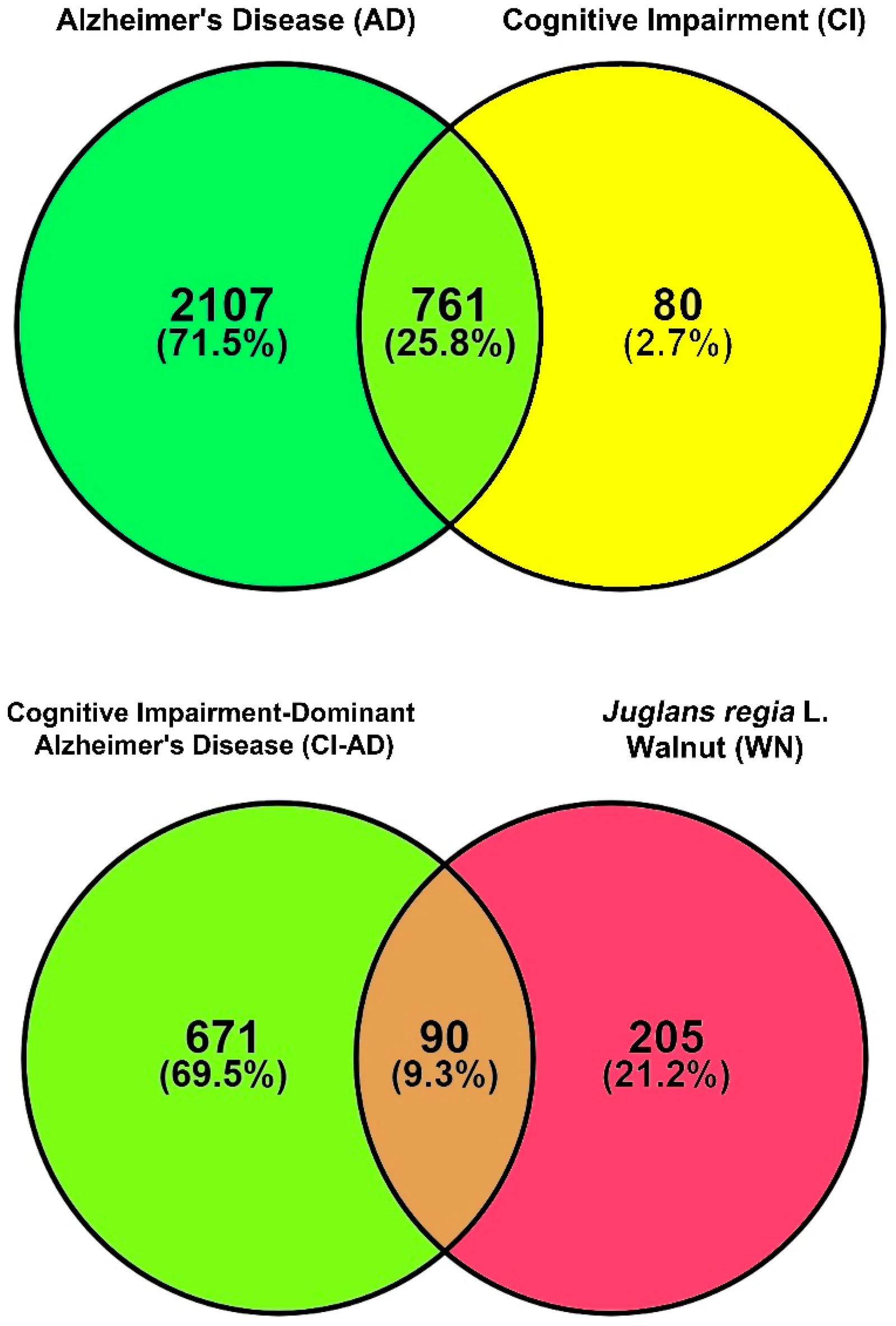
Identification of target gene screening. Venn diagram analysis illustrating the intersection of target genes. The upper panel shows the overlap between AD and CI, identifying 761 shared targets. The lower panel shows the intersection between CI-AD targets and WN predicted targets, revealing 90 core target proteins.

In this study, we characterized a diverse profile of 25 bioactive constituents derived from WN, comprising major chemical classifications such as fatty acids (e.g., alpha-linolenic acid, linoleic acid, and oleic acid), phenolic acids (e.g., gallic acid, syringic acid, and p-coumaric acid), and tannins (e.g., pedunculagin, tellimagrandin I, and strictinin) [19]. To strengthen the pharmacological rigor of our screening, the pharmacokinetic parameters, including the corresponding half-life time (t1/2) and lipophilicity (LogP) values for each metabolite, are integrated into Table S1. Furthermore, to address the practical relevance of these compounds in walnut kernels, we performed a comprehensive literature cross-verification of existing analytical evidence (e.g., HPLC-MS and GC-MS), which is systematically summarized in Table S2. Through screening across the TCMSP and ChEMBL databases, 295 candidate target proteins associated with these walnut constituents were retrieved. Concurrently, our disease-specific mining identified 2868 protein targets linked to AD and 841 targets linked to CI, establishing the basis for identifying 90 core shared genes involved in CI-AD pathogenesis. Venn diagram analysis (Figure 1, top) revealed a significant pathological overlap between AD and CI, with 761 shared genes accounting for 25.8% of the total identified disease targets. This substantial intersection underscores the highly conserved molecular pathways driving the transition from cognitive decline to clinical dementia. To isolate the most promising therapeutic targets, we intersected the walnut-associated proteins with the CI-AD gene set. This analysis revealed 90 core targets (9.3% overlap) that are simultaneously regulated by walnut constituents and involved in CI-AD pathogenesis (Figure 1, bottom). These 90 shared genes represent the primary pharmacological interface through which the multi-component matrix of WN—particularly its rich phenolic and fatty acid fractions—exerts its neuroprotective effects, providing a molecular basis for the systemic modulation of neurodegenerative cascades.

### 2.2. Compound–Target Network Construction and Systematic Correlation Analysis

**Figure 2:**
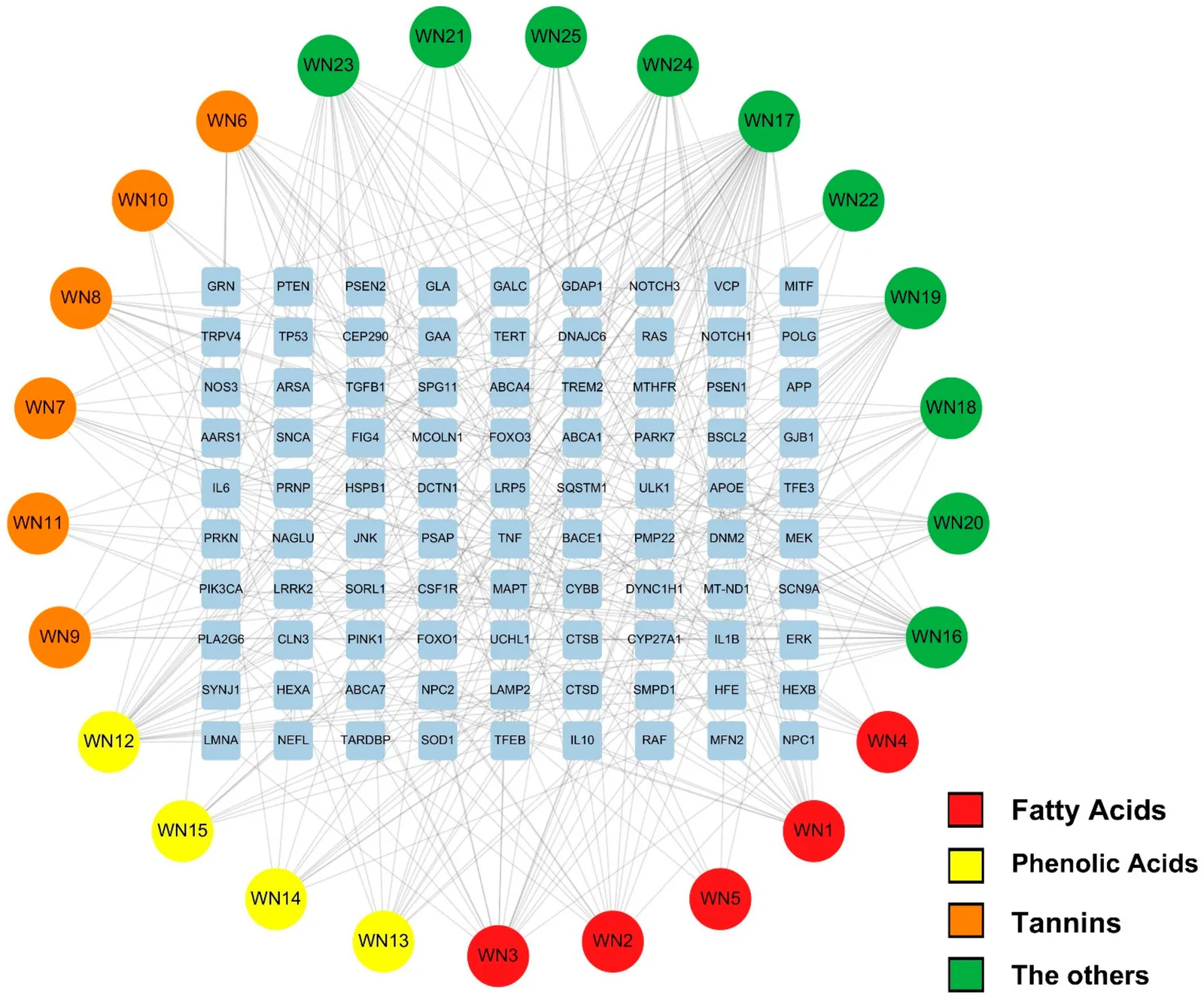
Compound–target network of walnut constituents in AD treatment. The network visualizes the complex pharmacological interactions between 25 bioactive walnut metabolites and their 90 core target genes. Circular nodes represent bioactive compounds, categorized by chemical class: red for fatty acids, yellow for phenolic acids, orange for tannins, and green for other bioactive groups. Central light-blue square nodes denote the 90 shared therapeutic targets. To enhance network readability, edge transparency has been optimized to highlight primary connections while reducing visual noise in the high-density interactome.

To provide a comprehensive visualization of the multi-component, multi-target regulatory landscape of WN, a complex C-T network was constructed. As illustrated in Figure 2, the network integrates the 25 bioactive constituents of walnut (labeled WN1 to WN25) with the 90 core therapeutic genes identified through our previous intersection analysis. The network architecture consists of 116 nodes and a dense array of edges representing the intricate correlations between phytochemicals and their neuroprotective targets. Within this interactome, the constituents are visually categorized by their chemical classes: fatty acids (red circles, WN1–WN5), phenolic acids (yellow circles, WN12–WN15), tannins (orange circles, WN6–WN11), and other bioactive groups including amino acids, tocopherols, and flavonoids (green circles, WN16–WN25). The central blue squares represent the core target genes, such as TFEB, mTOR, ABCA1, MAPK1, and APOE. The network topography reveals a high degree of connectivity, where multiple compounds typically converge on a single regulatory node, and, conversely, single compounds (such as gallic acid or pedunculagin) modulate multiple downstream proteins. This highly redundant and interconnected structure provides strong molecular evidence for the multi-component system of walnut extracts. Specifically, the widespread distribution of edges originating from the fatty acid and tannin fractions toward hub proteins involved in endolysosomal biogenesis and Aβ clearance suggests that these distinct chemical classes work in concert to restore neuronal proteostasis.

### 2.3. Construction and Analysis of the Protein–Protein Interaction Network

**Figure 3:**
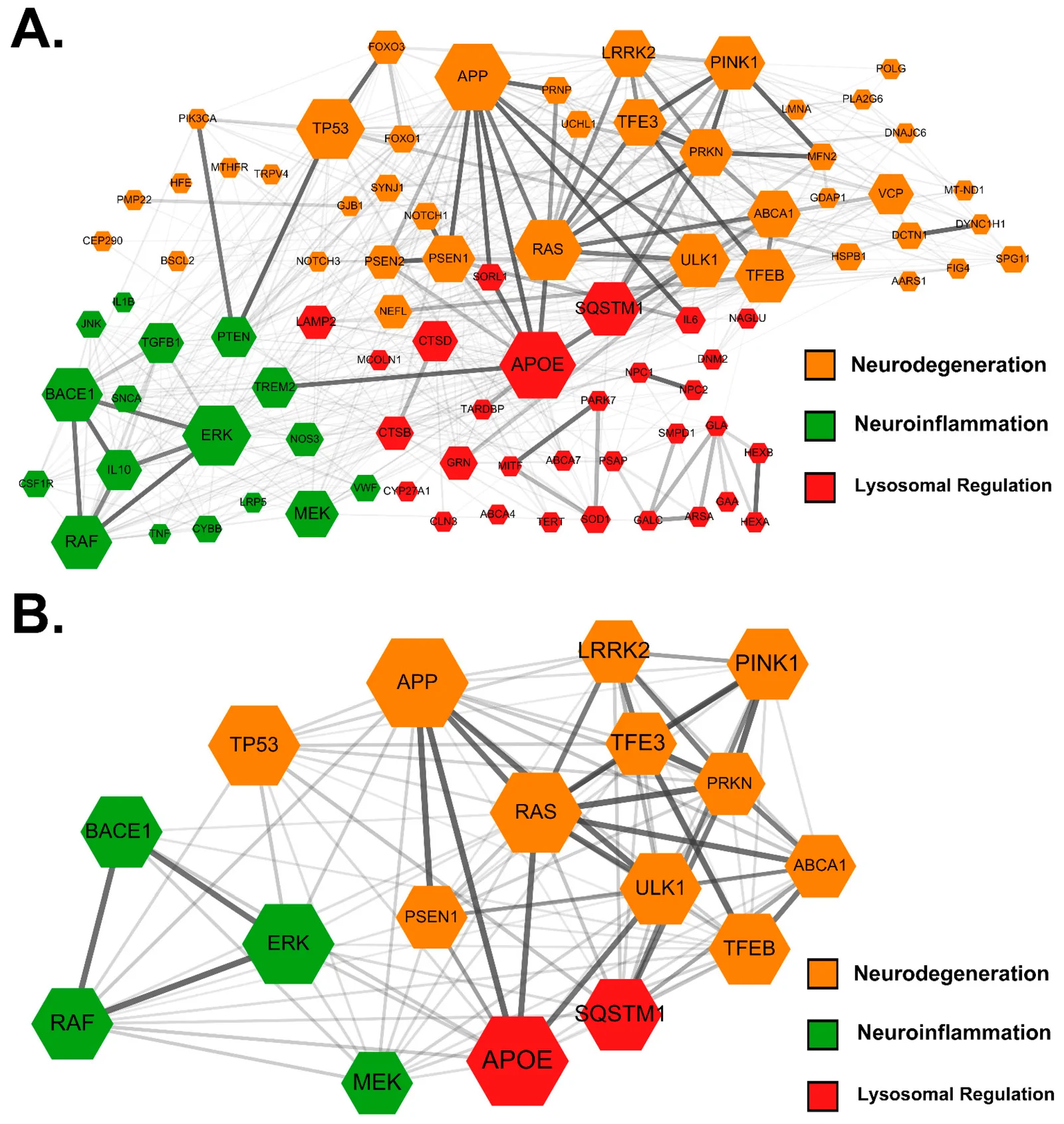
Protein–protein interaction network and functional cluster visualization. (**A**) Complete PPI network of the 90 identified targets. (**B**) Enlarged subpanel highlighting key hubs with degree > 23. Hexagonal nodes are color-coded by functional clusters: orange for neurodegeneration, green for neuroinflammation, and red for lysosomal regulation. Node size is proportional to degree centrality, emphasizing central hubs like APOE, APP, RAS, and TFEB. Edge thickness and transparency reflect the interaction confidence scores from the STRING database.

To elucidate the potential multi-target interactions and topological significance of the isolated therapeutic targets, we constructed a comprehensive PPI network using the 90 core genes. As shown in Figure 3, the resulting network is characterized by a high degree of interconnectivity, reflecting the systemic nature of AD pathogenesis and its predicted modulation by WN phytochemicals. The network is organized into three primary functional clusters, represented by distinct colors: neurodegeneration (orange hexagons), neuroinflammation (green hexagons), and lysosomal regulation (red hexagons). The size of each node reflects its relative degree centrality, while the thickness and transparency of the edges represent the strength of the interaction based on combined confidence scores. Topological analysis was conducted to identify the core hub proteins hypothesized to mediate the network’s regulatory functions. Based on stringent screening criteria (degree ≥ 23, BC ≥ 0.02, CC ≥ 0.5), we isolated several high-priority targets that occupy central positions within the interactome (Table S3). Notably, APOE (degree = 41, BC = 0.1232, CC = 0.6068) and APP (degree = 38, BC = 0.0514, CC = 0.5906) emerged as the primary hubs within the lysosomal and neurodegeneration clusters, respectively. Other critical regulators identified include RAS (degree = 33, BC = 0.0546), ERK (degree = 30, BC = 0.0275), and TFEB (degree = 29, BC = 0.0354), highlighting the central importance of the MAPK–mTORC1–TFEB axis in the predicted multi-component profile of WN. Furthermore, the presence of significant inter-cluster interactions suggests that the multi-component walnut matrix may simultaneously target multiple pathogenic domains, such as the synchronous suppression of neuroinflammation (via BACE1 and RAF) and the enhancement of proteostatic clearance (via SQSTM1 and ABCA1). This modular architecture provides a robust computational framework and an integrated mechanistic hypothesis for the potential neuroprotective effects of walnut-derived functional ingredients in restoring endolysosomal and synaptic homeostasis.

### 2.4. KEGG Pathway and GO Functional Enrichment Analysis

**Figure 4:**
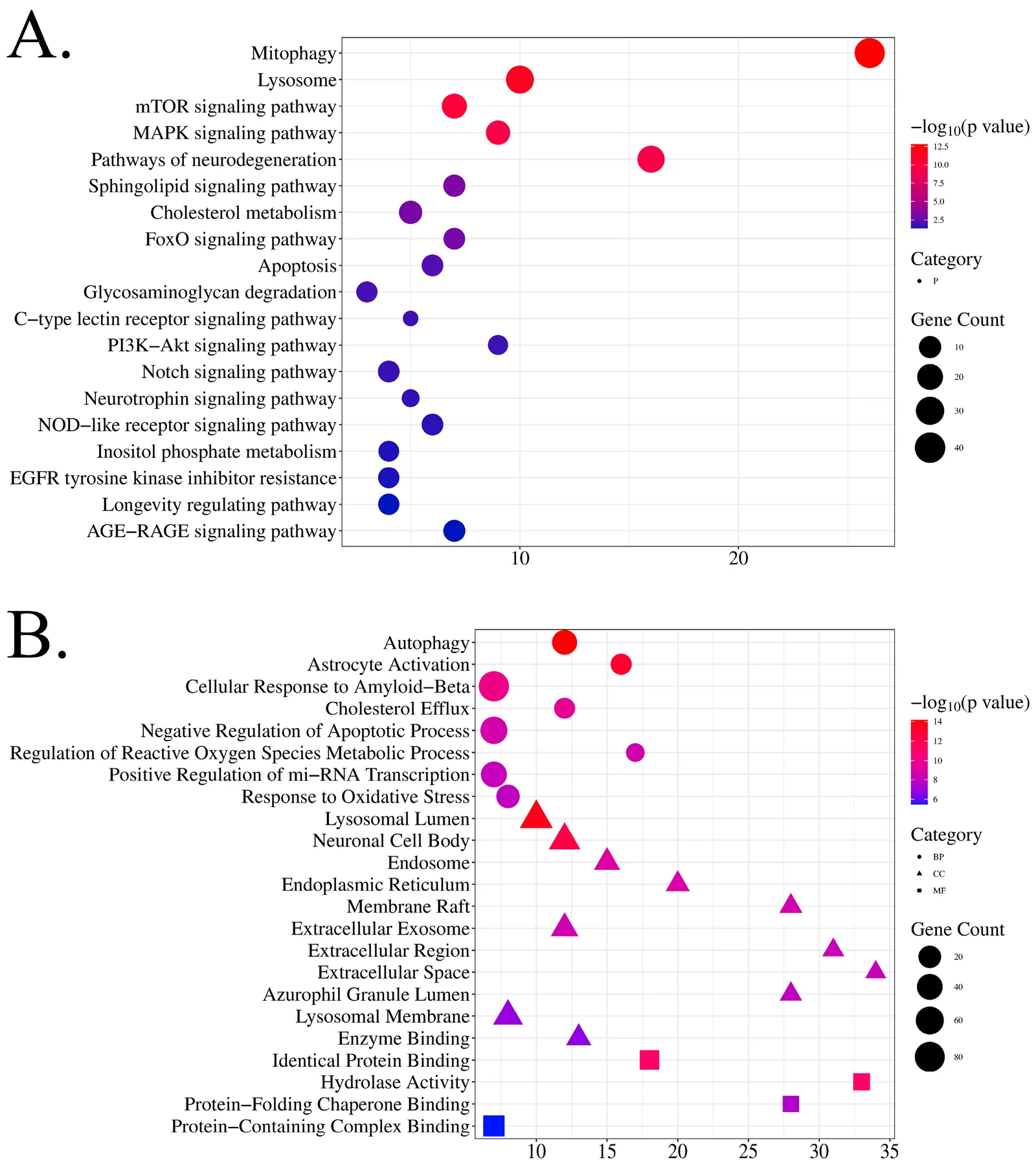
KEGG and GO enrichment analysis of the core therapeutic targets. (**A**) Bubble plot representing the KEGG pathway enrichment analysis. The *y*-axis denotes the categorical names of the enriched signaling pathways and metabolic processes. Bubble size indicates the gene count (*x*-axis); color indicates the statistical significance (−log *p* value). Key pathways include mitophagy, lysosome, and the MAPK/mTOR axis. (**B**) GO enrichment analysis categorized into biological process (BP), cellular component (CC), and molecular function (MF). The *y*-axis represents specific categorical biological terms. Node shapes distinguish the functional categories—circles for BP, triangles for CC, and squares for MF—with the *x*-axis representing the number of targets involved in each term.

To provide a high-resolution view of the molecular cascades modulated by WN, we performed KEGG pathway and GO functional enrichment analyses. As illustrated in Figure 4A and Table S4, KEGG analysis revealed a hierarchy of highly significant signaling axes dominated by proteostatic and metabolic homeostasis. Mitophagy (*p* = 1.71 × 10^−13^; count = 26) emerged as the most significantly enriched pathway with a fold enrichment (FE) of 42, followed by the lysosome pathway (*p* = 2.91 × 10^−12^; FE = 31), which includes 10 core targets. Importantly, the mTOR (*p* = 7.65 × 10^−11^; FE = 20) and MAPK (*p* = 2.53 × 10^−10^; FE = 17) signaling pathways exhibited strong statistical significance, supporting their roles as central regulatory nodes in JR-mediated neuroprotection. In addition, broader pathways such as pathways of neurodegeneration (*p* = 3.81 × 10^−10^; count = 16), along with lipid-related pathways including cholesterol metabolism (*p* = 9.92 × 10^−4^) and sphingolipid signaling (*p* = 6.70 × 10^−4^), suggest systemic modulation of membrane dynamics and lipid rafts, which are critical for Aβ processing. The GO enrichment analysis (Figure 4B and Table S5) further expanded these findings across three functional domains. Within the biological process (BP) category, targets were significantly enriched in autophagy (*p* = 6.90 × 10^−15^; FE = 40) and astrocyte activation (*p* = 8.40 × 10^−14^; FE = 15.6), indicating a dual regulatory role in enhancing cellular clearance while suppressing neuroinflammation. Notably, cellular response to Aβ exhibited the highest enrichment (*p* = 9.00 × 10^−11^; FE = 89.3), highlighting the specificity toward AD-associated pathology. In the cellular component (CC) category, enrichment was primarily localized to the lysosomal lumen (*p* = 2.50 × 10^−14^), endosome (*p* = 7.5 × 10^−10^), and endoplasmic reticulum (*p* = 1.1 × 10^−9^), outlining a complete intracellular transport-to-degradation continuum. Finally, in the molecular function (MF) category, hydrolase activity (*p* = 9.5 × 10^−12^; count = 33) and identical protein binding (*p* = 7.4 × 10^−12^) were significantly enriched, underscoring the regulation of enzymatic degradation and protein stability. Collectively, these results reveal that JR orchestrates a multi-layered therapeutic response, primarily through the MAPK–mTORC1–TFEB axis, to enhance lysosomal biogenesis, regulate lipid metabolism, and mitigate oxidative stress-induced neurodegeneration.

### 2.5. Integrative Pathway Model of the MAPK-mTORC1-TFEB Axis

**Figure 5:**
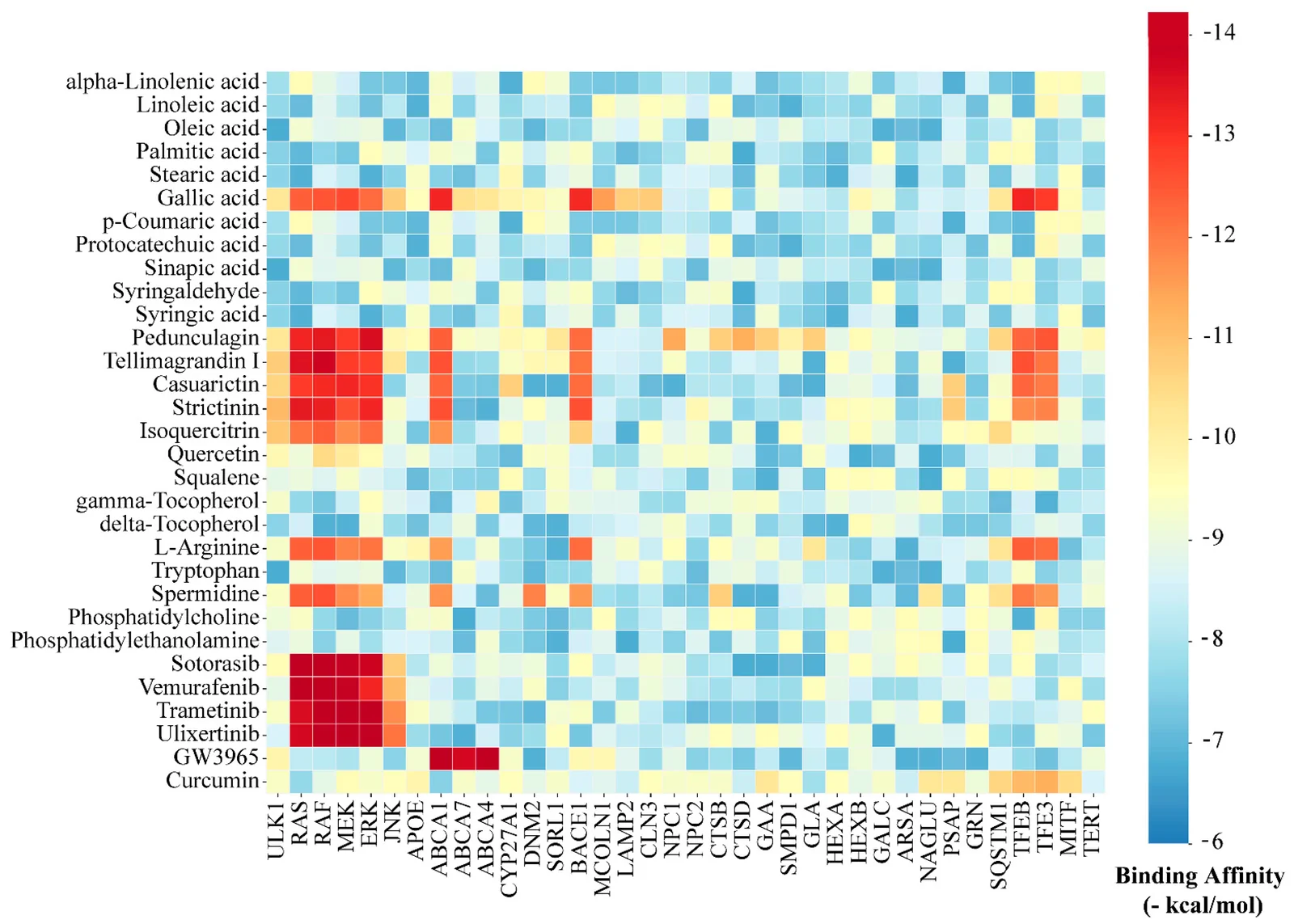
Heatmap of binding affinities between walnut constituents, positive control ligands, and core therapeutic targets. The color gradient represents the absolute binding energy (−kcal/mol). Six established pharmacological agents (Sotorasib, Vemurafenib, Trametinib, Ulixertinib, GW3965, and Curcumin) were integrated as positive controls to provide a quantitative benchmark for interaction strength. Dark-red regions indicate exceptionally strong affinities, prominently observed between the tannin fraction and the MAPK signaling cascade (RAS, RAF, MEK, and ERK), as well as between gallic acid and the TFEB/ABCA1 regulatory axis. Notably, several walnut-derived constituents reveal binding potencies within the same magnitude as these clinical reference inhibitors and putative regulators, supporting their potential as multi-target neuroprotective agents.

To evaluate the thermodynamic feasibility of the predicted interactions, we performed high-precision molecular docking on the pivotal nodes of the MAPK-mTORC1-TFEB regulatory axis. As illustrated in the comprehensive binding affinity heatmap (Figure 5 and Table S6), the bioactive constituents of WN exhibit a distinct, category-specific binding profile toward pro-pathogenic signaling proteins and lysosomal homeostasis regulators. To contextualize these findings, six established pharmacological agents were integrated as benchmarks: four targeted kinase inhibitors (Sotorasib—included as a structural reference for conserved RAS-family binding pockets—Vemurafenib, Trametinib, and Ulixertinib), the LXR agonist GW3965 (utilized as a structural reference for lipid-regulatory binding sites), and the BBB-permeable natural compound Curcumin. These results highlight a sophisticated dual-action mechanism where the larger ellagitannins act as putative upstream inhibitors, while smaller phenolic acids serve as downstream putative regulators of the endolysosomal system. Specifically, the tannin fraction revealed remarkable inhibitory potential against upstream pro-pathology kinases. Binding affinities for walnut constituents such as strictinin–RAS (−12.8 kcal/mol) and pedunculagin–RAF (−12.4 kcal/mol) were found to be within the same order of magnitude as the clinical benchmarks Sotorasib (−14.7 kcal/mol) and Vemurafenib (−13.8 kcal/mol), suggesting a robust capability to suppress hyperactive kinase cascades. In contrast, the smaller gallic acid molecule exhibited a highly specific and favorable binding affinity toward clearance core components, notably ABCA1 (−9.4 kcal/mol) and TFEB (−9.2 kcal/mol). This calculated binding profile aligns with our benchmark results for the lipid-regulatory reference GW3965 (−13.5 kcal/mol) and the TFEB reference Curcumin (−11.1 kcal/mol). The inclusion of Curcumin, a well-documented neuroprotective agent capable of crossing the blood–brain barrier, serves as a direct natural-product analog that reinforces the pharmacological relevance of walnut-derived metabolites in promoting lipid homeostasis and TFEB-mediated lysosomal biogenesis. It must be explicitly noted, however, that these computational comparisons are strictly theoretical. While such thermodynamic docking similarities offer valuable insights into structural compatibility and spatial positioning within binding pockets, they do not directly translate to equivalent experimental potency or functional biochemical activity, which necessitates future in vitro and in vivo validation.

**Figure 6:**
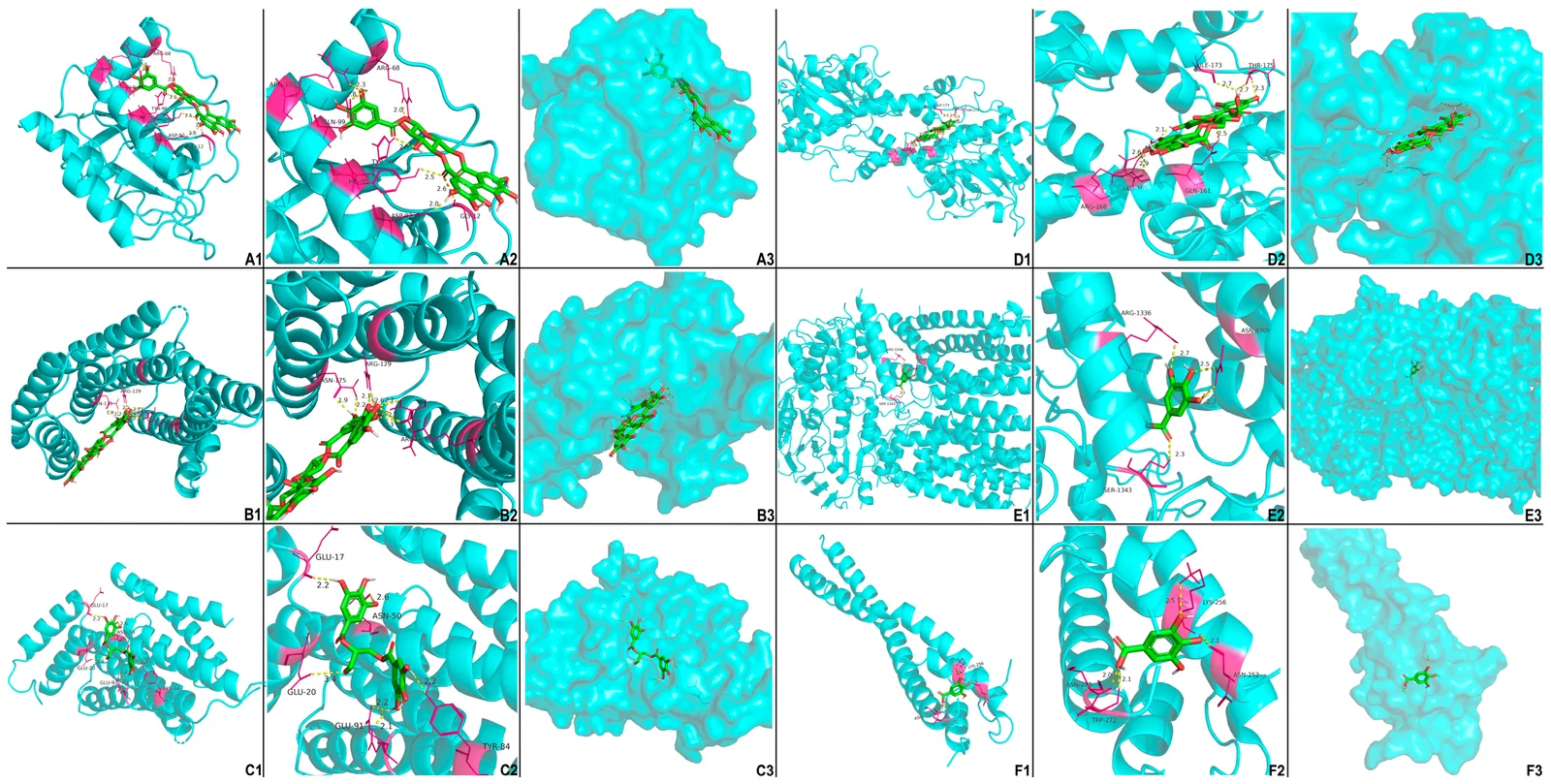
Molecular docking visualization of core compound–target interactions. (**A1**–**D3**) Predicted binding modes of tannins (strictinin, pedunculagin, tellimagrandin I) with RAS, RAF, and ERK, demonstrating steric pocket occupancy. (**E1**–**F3**) Precise docking conformations of gallic acid with ABCA1 and TFEB, highlighting stable polar hydrogen bonding within activation motifs. In all panels, the target proteins are colored in cyan, the active compounds are represented in green, key interacting amino acid residues are highlighted in magenta, and hydrogen bonds are depicted as yellow dashed lines.

Visual analysis of the three-dimensional docking conformations (Figure 6) reveals the predicted precise atomic-level interactions responsible for these high computational affinities. The complex hydrolyzable tannins, such as strictinin and pedunculagin, are shown to computationally establish dense networks of hydrogen bonds within the catalytic pockets of RAS, RAF, and ERK. These bulky ligands are predicted to occupy the ATP-binding sites and allosteric regulatory regions, theoretically arresting downstream signal transduction if physiologically available at the target site. Concurrently, gallic acid fits snugly into the activation motifs of TFEB and ABCA1, forming stable polar bonds with critical residues that are hypothesized to facilitate dephosphorylation and nuclear translocation, providing a rigorous computational rationale for the hypothesized multi-component profile of the walnut matrix.

**Figure 7:**
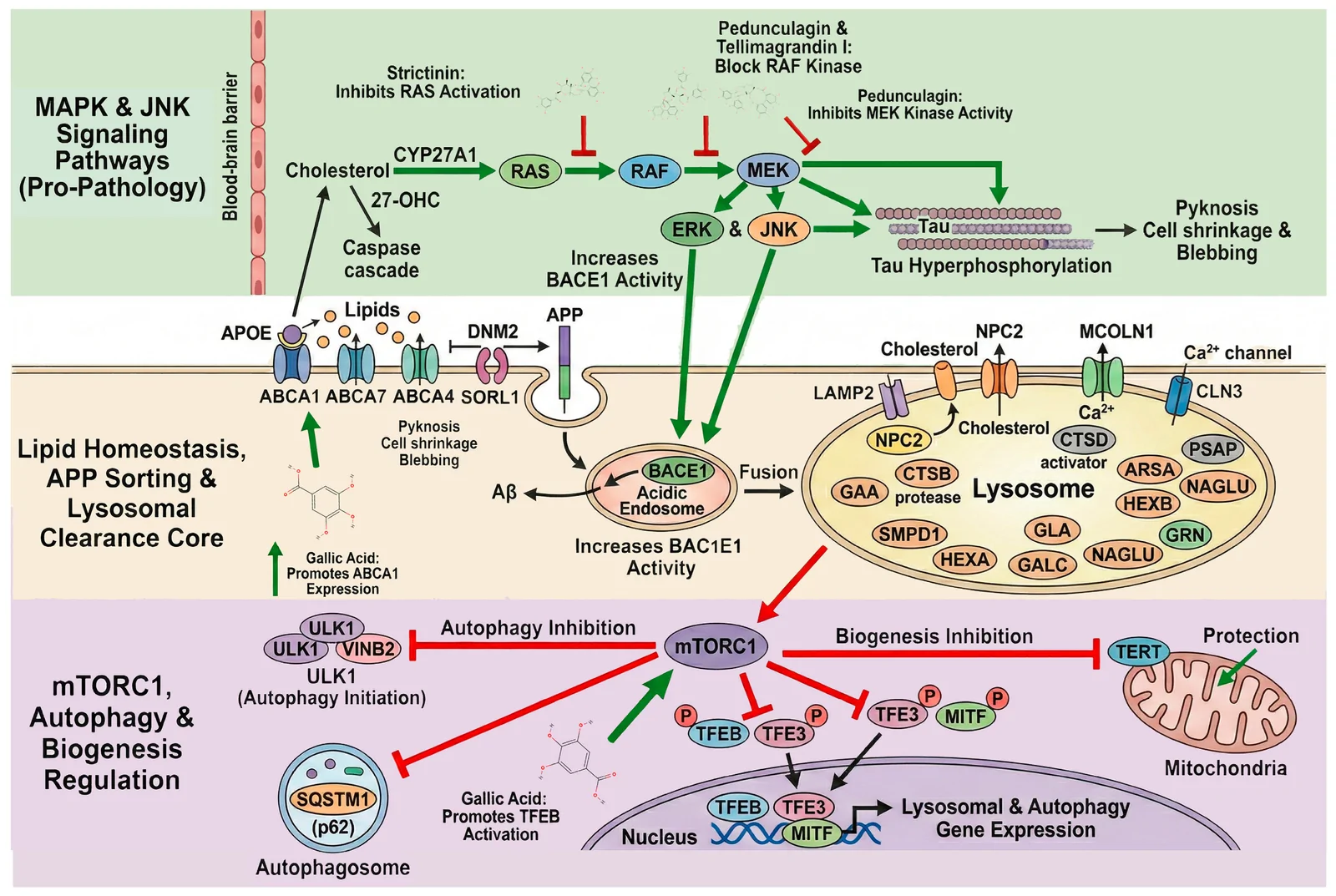
Predicted integrative pathway model of walnut-mediated neuroprotection. Integrative mechanistic pathway model of walnut-derived bioactives on the MAPK-mTORC1-TFEB axis in Alzheimer’s disease. This comprehensive signaling network delineates established canonical pathway relationships in contrast with the compound–target effects inferred from the present computational workflow. The network illustrates a hypothesized coordinated dual-action mechanism across three distinct cellular domains: the upper “Pro-Pathology” region (green) outlines the background MAPK/JNK signaling cascade as an established pathological axis, where Strictinin, Pedunculagin, and Tellimagrandin I are predicted to act as putative inhibitory “brakes” (red T-bars) on RAS, RAF, and MEK to mitigate Tau hyperphosphorylation and prevent pyknosis-induced cell shrinkage; the middle “Clearance Core” region (white) represents established homeostatic hubs for lipid transport (ABCA1, ABCA7, APOE) and APP sorting (SORL1, DNM2), onto which the modulatory effect of gallic acid is computationally mapped to enhance ABCA1 expression and facilitate endosome-to-lysosome fusion for toxic Aβ clearance; and the lower “Regulation” region (purple) reflects the established mTORC1-mediated control of autophagy and lysosomal biogenesis, where gallic acid is hypothesized to computationally overcome mTORC1-induced repression to trigger TFEB nuclear translocation, thereby activating the CLEAR gene network to restore lysosomal enzymatic activity (e.g., CTSB, CTSD, GAA) and mitochondrial health via TERT-mediated protection. This explicit integration ensures that the model is appropriately interpreted as an integrative computational hypothesis.

By integrating these docking insights with IPA-simulated signaling cascades, we developed a comprehensive integrative pathway model (Figure 7) that delineates the hypothesized systemic neuroprotective strategy of walnut bioactives. In this model, the MAPK braking effect is proposed to be achieved through the putative inhibition of the RAS-RAF-MEK-ERK signaling axis by tannins such as strictinin, pedunculagin, and tellimagrandin I, which is theoretically linked to reduced Tau hyperphosphorylation and the prevention of pyknosis-related cell shrinkage. Simultaneously, the endolysosomal acceleration is computationally modeled to be modulated by gallic acid, which putatively supports ABCA1 expression to maintain lipid homeostasis and facilitates TFEB activation. This predicted coordinated activation is hypothesized to overcome the biogenesis inhibition typically imposed by mTORC1, thereby potentially upregulating the Coordinated Lysosomal Expression and Regulation (CLEAR) gene network. This hypothesized dual modulation is predicted to support the acidic endosome-to-lysosome fusion process, theoretically ensuring the efficient clearance of Aβ aggregates and restoring mitochondrial health through TERT-mediated protection. Together, these results provide a robust computational foundation for WN as a multi-component functional food intervention with the theoretical potential to concurrently address pathogenic progression and enhance cellular clearance in AD, pending future experimental validation.

## 3. Discussion

The systematic characterization of the WN phytochemical matrix reveals a significant correlation between molecular structural descriptors and their respective neurotherapeutic potential in AD. Our computational findings, derived from high-resolution molecular docking and network pharmacology analyses, indicate that the ellagitannin fraction—comprising complex hydrolyzable tannins such as strictinin, pedunculagin, and tellimagrandin I—exhibits superior binding energies frequently exceeding −12.0 kcal/mol. These strong affinities are primarily attributed to the extensive hydrogen-bonding networks formed between the polyhydroxyl groups of tannins and the catalytic subdomains of pro-pathogenic kinases [20,21]. However, evaluation of their physicochemical properties reveals a potential translational limitation in terms of cerebral bioavailability. The lipophilicity (LogP) of these parent tannins typically falls outside the optimal range required for passive diffusion across the blood–brain barrier (BBB), which is generally considered to be between 1 and 5 for central nervous system (CNS)-active compounds [12,22,23]. Their high polar surface area (PSA) and suboptimal LogP values suggest that the observed in vivo neuroprotective effects of walnuts are unlikely to be mediated solely by the direct action of these large parent molecules. Instead, accumulating evidence indicates that these effects may be driven by systemic mechanisms, particularly the gut microbiota-mediated biotransformation of ellagitannins into smaller metabolites such as urolithins [24,25,26]. These metabolites exhibit more favorable lipophilicity and enhanced BBB permeability, thereby better satisfying the physicochemical requirements for central neuropharmacological activity.

The therapeutic potential of WN constituents represents a significant departure from conventional monotherapy, addressing the inherent “interconnectedness” of AD pathogenesis through the restoration of systemic proteostasis [27,28]. Unlike current Aβ-targeted monoclonal antibodies that focus on the sequestration of isolated protein aggregates—often resulting in dose-limiting side effects such as amyloid-related imaging abnormalities (ARIA)—the multi-component profile identified in this study is hypothesized to modulate the broader cellular environment [29,30]. When conceptualizing this profile, it is essential to distinguish between a botanical extract and traditional multi-component system. In contemporary medicinal chemistry, multi-component system often refers to the framework of Multi-Target-Directed Ligands (MTDLs), where a single synthesized molecule is rationally designed to interact with multiple specific targets simultaneously—a strategy recently exemplified by advanced MTDL developments for neurodegeneration [31]. In contrast, the present study investigates a multi-component matrix, wherein discrete phytochemical classes are independently associated with multiple predicted targets. Our PPI analysis, which highlights APOE, APP, and TFEB as central regulatory hubs, aligns with the high FE observed in mitophagy (FE = 42) and cellular response to Aβ (FE = 89.3). However, it must be emphasized that network density and enrichment analyses primarily support hypothesis generation. Therefore, rather than serving as definitive evidence of functional synergy, these topological results provide an integrated mechanistic hypothesis suggesting a potential fundamental re-programming of the neuronal degradative machinery [32,33]. Specifically, walnut-derived bioactives appear to circumvent the mTORC1-mediated biogenesis blockade, a pathogenic hallmark of AD that sequesters the master transcription factor TFEB in the cytoplasm [34,35,36]. By facilitating TFEB dephosphorylation and subsequent nuclear translocation, these compounds activate the CLEAR gene network, thereby restoring lysosomal acidification and enhancing the activity of critical hydrolases such as CTSB and CTSD [37,38].

A sophisticated mechanistic compartmentalization defines the hypothesized multi-component action of walnut phytochemical classes upon the MAPK-mTORC1-TFEB axis. At the atomic level, our docking simulations predict that large ellagitannins possess a high theoretical binding affinity within the upstream “Pro-Pathology” signaling cascade. However, consistent with their pharmacokinetic limitations, this predicted direct CNS interaction must be reconciled with physiological realities. Specifically, the profound inhibitory capacity calculated for these large tannins likely maps the pharmacophoric potential of the matrix, whereas their actual in vivo neuroprotection is putatively mediated by systemic anti-inflammatory effects or through their BBB-permeable circulating metabolites (e.g., urolithins). With this context in mind, when compared to the pharmacological gold standards of kinase inhibition, the theoretical inhibitory strength of walnut constituents closely rivals that of clinical agents. For instance, the binding affinity of tellimagrandin I (−13.8 kcal/mol) toward RAF occupies a thermodynamic range remarkably similar to that of the targeted covalent inhibitor Vemurafenib or Ulixertinib (−14.2 kcal/mol) [39,40,41]. Through significant steric blockade within the catalytic pockets of RAS, RAF, and MEK, these walnut ligands are predicted to structurally accommodate and theoretically attenuate the kinase cascades responsible for BACE1 upregulation and subsequent Tau hyperphosphorylation [11,42,43]. To contextualize these kinase comparisons from a structural biology standpoint, although Sotorasib is a mutation-specific inhibitor targeting KRAS G12C, its integration within our HRAS-based docking framework leverages the highly conserved three-dimensional architecture of the G-domain and its switch I/II regions across the broader RAS protein family, serving strictly as a structural reference for pocket accommodation. In parallel, the smaller phenolic acid fraction, exemplified by gallic acid, possesses favorable lipophilicity for direct CNS penetration and is predicted to function as a putative regulatory modulator of the endolysosomal clearance core [44,45]. Due to its lower molecular volume and optimized spatial orientation, gallic acid effectively targets the regulatory motifs of ABCA1 and TFEB [46,47,48]. This regulatory induction mirrors the activity of the synthetic LXR agonist GW3965 and the natural neuroprotective agent Curcumin. While GW3965 serves as a putative benchmark for ABCA1-mediated lipid homeostasis (−13.5 kcal/mol), gallic acid (−9.4 kcal/mol) exhibits a high-affinity profile comparable to Curcumin (−11.1 kcal/mol) [49]. The inclusion of Curcumin is particularly significant, as it represents a well-documented, BBB-permeable natural putative regulator that facilitates TFEB-mediated lysosomal biogenesis [50,51]. This interaction upregulates ABCA1-mediated lipid homeostasis and facilitates the biogenesis of functional lysosomes, thereby neutralizing the homeostatic inhibition typically maintained by the mTORC1 complex [52,53,54]. This bimodal regulatory strategy addresses AD pathology from both the biosynthetic synthesis and degradative clearance perspectives, providing a rigorous structural basis for the poly-pharmacological superiority of the walnut matrix. However, it must be explicitly emphasized that all comparative docking scores derived herein represent theoretical computational binding similarities and thermodynamic compatibility within structural pockets, rather than direct translations of experimental pharmacological potency or functional biochemical activity.

While these in silico results and the predicted thermodynamic stability provide a robust conceptual framework, aligning with contemporary paradigms that strongly advocate for integrating structure-based computational analyses with subsequent experimental pharmacology, several methodological constraints must be addressed in subsequent research [55]. The current study is primarily predicated on computational modeling, which necessitates empirical computational analysis through in vivo longitudinal studies using transgenic AD models, such as 5xFAD or APP/PS1 mice, to quantify cognitive preservation and synaptic plasticity improvements [56,57,58,59]. Furthermore, the kinetics of xenobiotic metabolism, particularly the conversion of ellagitannins into urolithin derivatives within the gut–brain axis, requires detailed pharmacokinetic profiling to indicate the concentration–time relationships within the CNS [60,61,62]. Although this study prioritized 90 core therapeutic targets, the holistic neuroprotective influence of WN likely involves secondary synergistic effects from minor constituents and trace nutrients that modulate the gut microbiome or systemic redox states. Future research utilizing integrative multi-omics platforms—incorporating transcriptomics and metabolomics—will be indispensable for bridging the gap between these systemic molecular insights and the development of evidence-based, multi-target dietary interventions for the management of neurodegenerative progression in the aging population.

## 4. Materials and Methods

### 4.1. Collection of Active Compounds in Juglans regia L. and Key Targets

To systematically elucidate the multi-target mechanisms of WN in the context of AD, we executed a comprehensive literature mining across digital repositories, including PubMed, ScienceDirect, and the Taiwan Electronic Theses and Dissertations System, documenting the phytochemical profiles alongside their analytical methodologies. These identified constituents were subsequently integrated into the TCM Systems Pharmacology (TCMSP, version 2.3) platform for pharmacokinetic evaluation, where a stringent ADME filtering strategy—requiring oral bioavailability (OB) ≥ 30% and drug-likeness (DL) ≥ 0.18—was applied to prioritize molecules with optimal pharmaceutical relevance [63]. Crucially, to ensure the natural occurrence and practical relevance of these screened compounds, we performed a literature-based analytical cross-verification. To efficiently manage the extensive literature and data during this phase, generative artificial intelligence Gemini (Google, https://gemini.google.com, accessed on 10 September 2026) was employed to assist in summarizing, comparing, and filtering the complex datasets of standardized pure components and their molecular targets. Only constituents with indicated evidence of being identified and quantified in walnut (*Juglans regia* L.) samples via independent HPLC-MS or GC-MS studies were retained for further analysis. The molecular targets for these screened compounds were predicted via the ChEMBL database, utilizing high-confidence filters (confidence score > 80%, activity threshold > 6) restricted to *Homo sapiens*, and subsequently standardized into official gene symbols through the UniProt Knowledgebase. Concurrently, AD-related genetic profiles were retrieved from GeneCards, the Therapeutic Target Database (TTD), and the Comparative Toxicogenomics Database (CTD) to define the disease-specific landscape [64]. To enhance the reliability of disease-associated targets, only GeneCards targets with a relevance score > 1 were retained [7]. All targets collected from different databases were standardized using official gene symbols, and duplicate entries were removed after standardization. Targets referring to the same gene or protein were merged into a single entry for subsequent analyses. By intersecting the walnut-associated targets with these AD-related genes and visualizing the overlap through Venn diagram analysis, we established a core gene set for subsequent systems biology investigation. Furthermore, Google Gemini was utilized to aid in the conceptualization and layout design of the graphical abstract. In strict accordance with journal guidelines, the authors rigorously reviewed, verified, and manually evaluated all AI-assisted data curation and visual outputs, taking full responsibility for the scientific accuracy and integrity of the final results.

### 4.2. Construction and Topological Analysis of Compound–Target Network and Protein–Protein Interaction Network

To visualize the complex interplay between walnut constituents and their corresponding targets, a C-T network was constructed using the Cytoscape software platform (version 3.9.1, USA). The systemic architecture of this network was evaluated by calculating key topological parameters, including degree centrality, betweenness centrality (BC), and closeness centrality (CC), which served as the primary indices for prioritizing the most topologically central candidate compounds. For the PPI analysis, the identified core targets were integrated into the STRING database (version 11.5), with the interaction environment restricted to *Homo sapiens* [65]. To ensure a balance between network depth and data reliability, we applied a high-confidence interaction score threshold of >0.7, effectively excluding isolated nodes with insufficient connectivity from the resulting interactome. Finally, the critical hub proteins hypothesized to mediate the neuroprotective effects were isolated through a stringent topological filtering process, utilizing thresholds of degree ≥ 23, BC ≥ 0.02, and CC ≥ 0.5 [66]. This multi-tiered analytical approach provided a computational framework to prioritize the central molecular nodes through which WN is predicted to modulate its multi-component regulatory functions in AD models, serving as the basis for our integrated mechanistic hypothesis [31].

### 4.3. Kyoto Encyclopedia of Genes and Genomes (KEGG) Pathway

To further elucidate the biological significance and pharmacological mechanisms underlying the identified core targets, we performed GO functional annotation and KEGG pathway enrichment analyses using the Database for Annotation, Visualization, and Integrated Discovery (DAVID, https://davidbioinformatics.nih.gov/). The GO analysis was categorized into three distinct domains—BP, CC, and MF—following the standardized classification of the GO Consortium. For the KEGG pathway enrichment, a threshold of raw *p* < 0.05 was initially established, followed by rigorous multiple testing correction using the Benjamini–Hochberg false discovery rate (FDR) procedure to ensure statistical robustness, with an adjusted significance threshold of FDR < 0.05 to identify statistically significant signaling cascades within the KEGG database associated with neurodegeneration [67,68]. Importantly, the results from these enrichment analyses were utilized to systematically classify the hub proteins identified in the previous PPI network analysis. By mapping the high-degree hub targets to specific enriched biological clusters, we categorized the core proteins into functional modules—such as neuroinflammation, Aβ clearance, and endolysosomal biogenesis. This integrated approach allowed us to correlate the topological importance of specific proteins with their definitive regulatory roles, offering a comprehensive understanding of how the bioactive components of WN orchestrate a coordinated, multi-target response against the pathogenic hallmarks of AD [69].

### 4.4. Ingenuity Pathway Analysis and Molecular Docking Computational Analysis

To simulate the regulatory cascades and systemic signaling pathways affected by walnut bioactives, we utilized the IPA software(QIAGEN, Redwood City, CA, USA; https://www.qiagen.com/us/products/discovery-and-translational-research/next-generation-sequencing/informatics-and-data/interpretation-content-databases/ingenuity-pathway-analysis, accessed on 30 June 2026). This platform allowed for the modeling of the MAPK-mTORC1-TFEB signaling axis, identifying upstream regulators and downstream effectors crucial for endolysosomal homeostasis [70]. A comprehensive panel of key hub proteins was investigated for their roles as upstream and downstream components within these pathways [71]. These proteins, acting as receptors, were subjected to molecular docking analysis to quantitatively assess their affinity with candidate walnut ligands.

Molecular docking was performed using AutoDock 4.2, employing the Lamarckian genetic algorithm. The three-dimensional structures of the target proteins were sourced from the RCSB PDB database [68,72]. Only protein models derived from X-ray crystallography or high-resolution cryo-EM with a resolution of less than 3 Å and an R-free value below 0.3 were selected, guaranteeing high crystal quality and model accuracy. Before docking, extraneous molecules such as antibody fragments, glycerol, water, free ions, and other non-relevant components were removed [73]. The selected receptors spanned several functional categories: upstream signaling kinases including ULK1 (PDB ID: 6QAT), HRAS (PDB ID: 4L9S), BRAF (PDB ID: 4RZV), MAP2K1 (PDB ID: 3EQC), MAPK1 (PDB ID: 6S9W), and MAPK8/JNK (PDB ID: 3ELJ); lipid and Aβ metabolism regulators such as APOE (PDB ID: 1GS9), ABCA1 (PDB ID: 5XJY), ABCA7 (PDB ID: 7X5V), ABCA4 (PDB ID: 7Z9Z), CYP27A1 (PDB ID: 2YOT), SORL1 (PDB ID: 7P0S), and BACE1 (PDB ID: 1SGZ); endolysosomal structural and transport proteins including DNM2 (PDB ID: 2PV2), MCOLN1 (PDB ID: 5WIV), LAMP2 (PDB ID: 5V14), NPC1 (PDB ID: 5U74), NPC2 (PDB ID: 2HKA), and SQSTM1 (PDB ID: 4X98); lysosomal enzymes such as CTSB (PDB ID: 1HVB), CTSD (PDB ID: 4OD9), GAA (PDB ID: 5NNJ), SMPD1 (PDB ID: 5I81), GLA (PDB ID: 3GZ1), HEXA (PDB ID: 2GJX), HEXB (PDB ID: 1NOU), GALC (PDB ID: 3ZR5), ARSA (PDB ID: 1AUK), NAGLU (PDB ID: 4XWH), PSAP (PDB ID: 4XU1), and GRN (PDB ID: 6Z2U); and key transcription factors/regulators including TFEB (PDB ID: 6U9A), TFE3 (PDB ID: 6U9B), MITF (PDB ID: 4X7V), and TERT (PDB ID: 7XKR). The docking site for each receptor was defined by a grid box of 60 × 60 × 60 points with a spacing of 0.375 Å, centered on the coordinates of the original co-crystallized ligands to target the orthosteric binding regions. To enhance the exhaustiveness and reliability of the search, 50 independent docking runs were performed for each ligand–protein pair, with the maximum number of energy evaluations set to 2.5 × 10^6^ per run.

Docking simulations were conducted under default parameters, generating up to nine binding modes for each protein–ligand interaction [74]. The binding mode with the highest absolute binding energy (lowest Gibbs free energy) was selected as the most favorable representation of receptor–ligand interaction. To evaluate the relative binding compatibility of the screened walnut constituents, six established pharmacological agents were utilized as structural and thermodynamic reference benchmarks. These included four targeted kinase inhibitors (Sotorasib—included as a structural reference for conserved RAS-family binding pockets rather than mutation-specific targeting—Vemurafenib, Trametinib, and Ulixertinib), the LXR agonist GW3965 (utilized as a reference for lipid-regulatory binding sites), and the BBB-permeable TFEB putative regulator Curcumin. All control ligands were subjected to the same docking protocols using AutoDock 4.2.6 to ensure a standardized thermodynamic comparison between the walnut phytochemicals and established molecules. It is explicitly noted that these benchmark comparisons are strictly theoretical and serve to evaluate computational binding similarities within defined structural pockets rather than validated experimental potencies. This high-precision computational approach provided a thermodynamic foundation for generating integrative mechanistic hypotheses regarding the multi-component profile of WN.

## 5. Conclusions

In summary, this in silico study proposes a comprehensive systems-level mechanistic hypothesis for the neuroprotective potential of WN by delineating a critical therapeutic axis centered on MAPK-mTORC1-TFEB-mediated endolysosomal homeostasis. Our computational findings suggest that the multi-component efficacy of the walnut phytochemical matrix stems from a sophisticated mechanistic compartmentalization: large hydrolyzable ellagitannins are predicted to provide inhibitory modulation of upstream pro-pathogenic signaling (RAS/RAF/MEK) to suppress Tau hyperphosphorylation and amyloidogenesis, while smaller phenolic acids, such as gallic acid, act as high-affinity putative regulators of the cellular clearance core via ABCA1 and TFEB motifs. This hypothesized dual-action mechanism computationally circumvents the mTORC1-mediated biogenesis blockade, potentially triggering the CLEAR gene network to restore lysosomal acidification and autophagic flux. By addressing AD pathology from both biosynthetic and degradative perspectives, this study offers a rigorous structural and energetic explanation for the multi-target superiority of the walnut botanical matrix. Ultimately, these results establish a robust computational foundation for walnuts as a multi-target functional food intervention, offering innovative avenues for integrated drug–food strategies in the global management of neurodegenerative diseases, which warrant future in vitro and in vivo experimental validation.

## Data Availability

Publicly available datasets were analyzed in this study. Phytochemical profiles and ADME properties were retrieved from TCMSP (https://tcmsp-e.com/) and ChEMBL (https://www.ebi.ac.uk/chembl/); disease-associated targets were integrated from GeneCards (https://www.genecards.org/), TTD (https://db.idrblab.net/ttd/), and CTD (https://ctdbase.org/); protein interaction and functional enrichment data were sourced from STRING (https://string-db.org/) and DAVID (https://davidbioinformatics.nih.gov/workspace.html?tool=summary_ws); and protein crystal structures were obtained from the RCSB PDB (https://www.rcsb.org/), all accessed on 8 September 2026. All other supporting data are included within the article and its Supplementary Materials.

## Supplementary Materials

The following supporting information can be downloaded at https://www.mdpi.com/article/10.3390/ijms27188288/s1. References [75,76,77,78,79,80,81,82,83,84] are cited in the Supplementary Materials.

## Abbreviations

AD	Alzheimer’s Disease
Aβ	Amyloid-Beta
TCM	Traditional Chinese Medicine
MAPK	Mitogen-Activated Protein Kinase
mTORC1	Mechanistic Target of Rapamycin Complex 1
TFEB	Transcription Factor EB
RAS	Rat Sarcoma Virus
RAF	Rapidly Accelerated Fibrosarcoma
ERK	Extracellular Signal-Regulated Kinase
PPI	Protein–Protein Interaction
GO	Gene Ontology
KEGG	Kyoto Encyclopedia of Genes and Genomes
IPA	Ingenuity Pathway Analysis
ADME	Absorption, Distribution, Metabolism, and Excretion
OB	Oral Bioavailability
DL	Drug-Likeness
HL	Biological Half-Life
TCMSP	Traditional Chinese Medicine Systems Pharmacology
TTD	Therapeutic Target Database
CTD	Comparative Toxicogenomics Database
STRING	Search Tool for the Retrieval of Interacting Genes/Proteins
DAVID	Database for Annotation, Visualization, and Integrated Discovery
PDB	Protein Data Bank
C-T	Compound–Target
BC	Betweenness Centrality
CC	Closeness Centrality
Cryo-EM	Cryogenic Electron Microscopy
ChEMBL	Chemical Database of Bioactive Molecules
WN	Walnut (*Juglans regia* L.)
FE	Fold Enrichment
JNK	c-Jun N-Terminal Kinase
APP	Amyloid Precursor Protein
APOE	Apolipoprotein E
ABCA1	ATP-Binding Cassette Transporter A1
BACE1	Beta-Site APP Cleaving Enzyme 1
SQSTM1	Sequestosome 1 (p62)
TERT	Telomerase Reverse Transcriptase
CTSB/CTSD	Cathepsin B/Cathepsin D
GAA	Alpha-Glucosidase
SORL1	Sortilin-Related Receptor 1
DNM2	Dynamin 2
CNS	Central Nervous System
ARIA	Amyloid-Related Imaging Abnormalities
BBB	Blood–Brain Barrier
PSA	Polar Surface Area
