# Supplementary material for: Exploring the Neuroprotective Effects of Walnut (Juglans regia L.) Against Cognitive Impairment in Alzheimer’s Disease Through the MAPK-mTORC1-TFEB-Mediated Endolysosomal Pathway: A Network Pharmacology and Molecular Docking Approach
Source: Int J Mol Sci. 2026 Sep 17;27(18):8288. doi: 10.3390/ijms27188288 (PMC13607112; doi:10.3390/ijms27188288)
Supplement: Supplementary file 1 [file ijms-27-08288-s001.zip › ijms-4548054-supplementary.pdf]

No.	Molecule Name	Classification	MW	OB (%)	DL	$t_{1/2}$ (h)	Lipophilicity (LogP)	Target Gene
WN1	alpha-Linolenic acid	Fatty Acids	278.43	80.5	0.43	14.3-16.1	3.3	TNF, IL6, IL1B, IL10, TGFB1, TREM2, CSF1R, NOS3, RAS, APOE, ABCA1, ABCA7, NPC1, NPC2, CYP27A1, PLA2G6, NOTCH3, GJB1, GDAP1
WN2	Linoleic acid	Fatty Acids	280.45	79.3	0.34	16.2-17.5	4	TNF, IL6, IL1B, IL10, TGFB1, NOS3, RAS, APOE, ABCA1, ABCA7, NPC1, NPC2, CYP27A1, PLA2G6
WN3	Oleic acid	Fatty Acids	282.46	76.4	0.45	3.3-4.1	4	TNF, IL6, IL1B, IL10, TGFB1, NOS3, APOE, ABCA1, ABCA7, NPC1, NPC2, PLA2G6, SCN9A, GJB1, PMP22, BSCL2, LMNA, NOTCH1, LRP5
WN4	Palmitic acid	Fatty Acids	256.42	83.3	0.35	4.1.-5.2	3.5	TNF, IL6, IL1B, CYBB, MEK, TP53, PTEN, PIK3CA
WN5	Stearic acid	Fatty Acids	284.48	70.2	0.64	4.8-5.7	4.5	TNF, IL6, IL1B, CYBB, MEK, TP53
WN6	Gallic acid	Phenolic Acids	170.12	38.1	0.24	1.6-.2.2	0.7	TNF, IL6, IL1B, IL10, TGFB1, CYBB, MEK, NOS3, PIK3CA, PTEN, FOXO1, FOXO3, TP53, BACE1, APP, HFE, UCHL1
WN7	p-Coumaric acid	Phenolic Acids	164.16	31.4	0.03	1.7-2.1	1.5	TNF, IL6, IL1B, CYBB, NOS3, PIK3CA, PTEN, FOXO1, FOXO3, TRPV4, JNK
WN8	Protocatechuic acid	Phenolic Acids	154.12	29.6	0.28	1.8-2.4	0.9	TNF, IL6, IL1B, IL10, TGFB1, CYBB, MEK, PIK3CA, PTEN, FOXO3, HSPB1, CEP290, MTHFR, TERT
WN9	Sinapic acid	Phenolic Acids	224.21	35.1	0.25	2.5-3.1	1.3	TNF, IL6, IL1B, CYBB, MEK, FOXO1, FOXO3, TRPV4
WN10	Syringaldehyde	Phenolic Acids	182.17	25.6	0.04	1.7-2.0	1.2	TNF, IL6, IL1B, CYBB, PIK3CA
WN11	Syringic acid	Phenolic Acids	354.31	80.2	0.24	1.9-2.4	1	TNF, IL6, IL1B, IL10, TGFB1, CYBB, PIK3CA
WN12	Pedunculagin	Tannins	180.16	25.8	0.03	3.2-3.9	-0.3	TFEB, TFE3, MITF, SQSTM1, LAMP2, MCOLN1, CLN3, CTSB, CTSD, GAA, GLA, HEXA, HEXB, NAGLU, ARSA, GALC, PSAP, SMPD1, GRN, TREM2, TNF, IL6, SPG11, FIG4
WN13	Tellimagrandin I	Tannins	194.18	26.1	0.23	2.8-3.3	0.3	TFEB, SQSTM1, LAMP2, CTSB, CTSD, PSAP, SMPD1, GRN, TREM2, TNF, IL6, IL1B, FIG4

WN14	Casuarictin	Tannins	168.15	25.9	0.32	2.4-3.1	1.1	TFEB, TFE3, SQSTM1, LAMP2, CTSB, CTSD, HEXA, HEXB, TNF, IL6, SPG11
WN15	Strictinin	Tannins	198.17	27.2	0.21	3.1-3.8	0.8	TFEB, MITF, SQSTM1, LAMP2, CTSB, CTSD, TREM2, TNF, IL6
WN16	Isoquercitrin	Flavonoids	464.38	40.2	0.24	2.2-2.9	-0.1	APP, PSEN1, PSEN2, BACE1, MAPT, SORL1, APOE, ABCA1, ABCA7, SNCA, LRRK2, PARK7, TNF, IL6, IL1B, IL10, TGFB1, PIK3CA, PTEN, FOXO1, FOXO3, TFEB, SQSTM1, CTSB, CTSD, DNM2, DNAJC6, SYNJ1
WN17	Quercetin	Flavonoids	302.24	28.9	0.03	11.5-12.1	1.5	APP, PSEN1, PSEN2, BACE1, MAPT, SORL1, APOE, ABCA1, ABCA7, SNCA, LRRK2, PRNP, VCP, TARDBP, PARK7, TNF, IL6, IL1B, IL10, TGFB1, TREM2, CSF1R, RAF, PIK3CA, PTEN, FOXO1, FOXO3, TP53, TFEB, SQSTM1, LAMP2, CTSB, CTSD, GRN, DYNC1H1, DCTN1, NEFL, DNM2, DNAJC6, SYNJ1, UCHL1, NOTCH1, NOTCH3, LRP5, POLG, TERT, LMNA, AARS1, CEP290, MTHFR, HFE, SOD1
WN18	Squalene	Triterpenes	410.72	60.3	0.13	12.2-13.4	8	APOE, ABCA1, ABCA7, ABCA4, NPC1, NPC2, CYP27A1, TNF, IL6, IL1B, CYBB, MEK, MT-ND1
WN19	gamma-Tocopherol	Tocopherols	416.69	54.4	0.32	12.8-14.4	7.5	TNF, IL6, IL1B, IL10, TGFB1, CYBB, MEK, NOS3, APOE, ABCA1, RAF, PARK7, DYNC1H1, NEFL, UCHL1, GJB1, PMP22, BSCL2, GDAP1, SOD1, POLG, MT-ND1, AARS1, MTHFR, HFE
WN20	delta-Tocopherol	Tocopherols	402.66	53.4	0.21	12.5-13.4	7	TNF, IL6, IL1B, CYBB, MEK, NOS3, APOE, DCTN1, SCN9A, SOD1
WN21	L-Arginine	Amino Acids	174.2	70.3	0.03	1.8-2.2	-4.2	NOS3, RAS, VWF, ERK, JNK, TNF, IL6, IL1B, TGFB1
WN22	Tryptophan	Amino Acids	204.23	80.4	0.28	2.3-2.9	-1.1	TNF, IL6, IL1B, IL10, TGFB1
WN23	Spermidine	Amino Acids	145.25	72.3	0.25	1.7-2.1	-1	TFEB, TFE3, MITF, SQSTM1, LAMP2, CTSB, CTSD, PINK1, PRKN, MFN2, POLG, MT-ND1, PARK7, FOXO3, TP53, TERT, LMNA,

								SPG11, FIG4, HSPB1, AARS1, CEP290, NEFL, GDAP1
WN24	Phosphatidylcholine	Phospholipids	414.71	79.3	0.04	17.9-19.2	8	NPC1, NPC2, APOE, ABCA1, ABCA7, ABCA4, CYP27A1, PLA2G6, APP, SORL1, TFEB, LAMP2, DNM2, SYNJ1, PMP22, BSCL2, LRP5
WN25	Phosphatidylethanolamine	Phospholipids	400.68	83.5	0.24	20.8-22.1	7.5	NPC1, NPC2, APOE, ABCA1, PLA2G6, TFEB, SQSTM1, LAMP2, DNAJC6

Table S1. The corresponding pharmacokinetic information and potential target genes of active compounds in WN against AD-CI.

No.	Compound	Literature Source	Extraction Method	Analytical Tool & Parameters
WN1	alpha-Linolenic acid	Savage, G. P. (2001). Chemical composition of walnuts (*Juglans regia* L.) grown in New Zealand. *Plant Foods for Human Nutrition*, 56(1), 75-82.	Soxhlet extraction with n-hexane or cold-pressing of the kernel.	GC-FID; Column: DB-23 (30m x 0.25mm); Temp prog: 150°C to 230°C at 3°C/min.
WN2	Linoleic acid	Ojeda-Amador, R. M., et al. (2018). Characterization of virgin walnut oils (*Juglans regia* L.) from Spanish cultivars. *Food Research International*, 110, 390-398.	Cold-pressing or Folch method (Chloroform/MeOH 2:1).	GC-MS; FAMEs derivation (KOH/MeOH); Injector: 250°C; Split ratio: 1:50.
WN3	Oleic acid	Gao, P., et al. (2018). Comparison of the chemical composition of walnut oils from different regions. *Journal of Food Composition and Analysis*, 74, 11-17.	Solvent extraction using petroleum ether.	GC-FID; Column: HP-88 (100m); Carrier: Helium (1.0 mL/min).
WN4	Palmitic acid	Tapia, M. I., et al. (2013). Determinants of the antioxidant capacity of pine nut and walnut oil. *Food Chemistry*, 138(2-3), 1652-1659.	Hexane dilution of oil samples.	GC-MS; Ion source temp: 230°C; MS Scan: m/z 50-550.
WN5	Stearic acid	Bada, J. C., et al. (2010). Fatty acid composition of walnuts from Asturias (Spain). *Food Chemistry*, 123(2), 304-307.	Direct transesterification with Methanol/H2SO4.	GC-FID; HP-Innowax column; Comparison with FAME Mix standard.
WN6	Gallic acid	Grace, M. H., et al. (2014). Comparative analysis of phenolic constituents and antioxidant capacity of different walnut cultivars. *Food Chemistry*, 156, 267-272.	Sonication with 80% Methanol.	HPLC-DAD; C18 Column; Mobile Phase: 0.1% Formic acid/ACN; Detection: 280 nm.
WN7	p-Coumaric acid	Slatnar, A., et al. (2015). Identification and quantification of phenolic compounds in walnuts. *Food Chemistry*, 173, 270-277.	Methanol/Water/HCl (80:19:1) extraction.	HPLC-DAD; Gradient elution (H2O/ACN); Detection: 320 nm.
WN8	Protocatechuic acid	Regueiro, J., et al. (2014). Comprehensive identification of walnut polyphenols by LC-LTQ-Orbitrap MS. *Food Chemistry*, 152, 340-348.	Homogenization in 70% Acetone/Water.	UHPLC-MS/MS; Negative mode ESI; Fragment ion: m/z 153.01.
WN9	Sinapic acid	Vu, D. C., et al. (2018). Identification and quantification of phytosterols and tocopherols in walnut. *Journal of Food Science*, 83(10), 2420-2427.	80% Ethanol extraction followed by hydrolysis.	HPLC-MS/MS; MRM mode; Q1/Q3 transition: 223/164.
WN10	Syringaldehyde	Grace, M. H., et al. (2014). *Food Chemistry*, 156, 267-272.	Liquid-liquid extraction with Ethyl Acetate.	HPLC-DAD; Zorbax SB-C18 column; Detection: 280 nm.

WN11	Syringic acid	Slatnar, A., et al. (2015). *Food Chemistry*, 173, 270-277.	Acidified Methanol extraction.	HPLC-DAD; Column: Gemini C18 (150 x 4.6mm); Detection: 280 nm.
WN12	Pedunculagin	Regueiro, J., et al. (2014). *Food Chemistry*, 152, 340-348.	70% Acetone/Water extraction (optimized for tannins).	LC-ESI-MS/MS; m/z 783.07 [M-H]-; MS2 fragments at m/z 481, 301.
WN13	Tellimagrandin I	Grace, M. H., et al. (2014). *Food Chemistry*, 156, 267-272.	Cold extraction with 80% MeOH.	LC-MS; Negative ion mode m/z 785; UV Detection: 254 nm.
WN14	Casuarictin	Regueiro, J., et al. (2014). *Food Chemistry*, 152, 340-348.	Solid Phase Extraction (SPE) cleanup.	UHPLC-Q-Exactive MS; Exact mass: 935.0831 [M-H]-.
WN15	Strictinin	Vu, D. C., et al. (2018). *Journal of Food Science*, 83(10), 2420-2427.	Sonication in Methanol.	LC-TOF-MS; m/z 633.07 [M-H]-; Typical RT before Pedunculagin.
WN16	Isoquercitrin	Grace, M. H., et al. (2014). *Food Chemistry*, 156, 267-272.	80% Aqueous Methanol.	HPLC-DAD; Detection: 360 nm; Standard calibration curve verification.
WN17	Quercetin	Slatnar, A., et al. (2015). *Food Chemistry*, 173, 270-277.	Acid hydrolysis (2M HCl) of flavonoids.	HPLC-DAD; Column: Gemini C18; Detection: 360 nm.
WN18	Squalene	Savage, G. P. (2001). *Plant Foods for Human Nutrition*, 56(1), 75-82.	Extraction of unsaponifiable matter with Diethyl Ether.	GC-MS; Column: HP-5; Oven: 200°C to 300°C; MS fragment: m/z 69.
WN19	gamma-Tocopherol	Ojeda-Amador, R. M., et al. (2018). *Food Research International*, 110, 390-398.	Direct injection of oil diluted in Hexane.	NP-HPLC; Column: Silica 60; FLD: Ex 290nm / Em 330nm.
WN20	delta-Tocopherol	Gao, P., et al. (2018). *Journal of Food Composition and Analysis*, 74, 11-17.	Cold-pressing extraction.	HPLC-UV; Column: C18; Mobile Phase: MeOH (100%); Detection: 292 nm.
WN21	L-Arginine	Cordeiro, M., et al. (2014). Amino acid profile of walnut cultivars. *Food Science and Technology*, 34(3), 562-567.	6M HCl hydrolysis at 110°C for 24h.	HPLC-FLD; Pre-column derivatization with OPA; Column: Spherisorb C18.
WN22	Tryptophan	Cordeiro, M., et al. (2014). *Food Science and Technology*, 34(3), 562-567.	Alkaline hydrolysis (NaOH) to prevent degradation.	HPLC-FLD; Ex 280nm / Em 340nm; Isocratic: 50mM NaOAc pH 4.3.
WN23	Spermidine	Cordeiro, M., et al. (2014). Polyamine content in walnut kernels.	Perchloric acid (5%) extraction.	HPLC-UV; Derivatization with Dansyl Chloride; Detection: 254 nm.
WN24	Phosphatidylcholine	Verardo, V., et al. (2013). Optimization of phospholipid extraction from walnut. *Food Chemistry*, 141(3), 2114-2122.	Chloroform/Methanol (2:1) - Folch method.	HPLC-ELSD or LC-MS; Column: Diol or HILIC; Mobile: ACN/H2O/NH4OAc.
WN25	Phosphatidylethanolamine	Verardo, V., et al. (2013). *Food Chemistry*, 141(3), 2114-2122.	Ultrasonic-assisted extraction in Chloroform.	LC-MS/MS; Positive ESI mode; Diagnostic fragment: m/z 141 (Phosphoethanolamine).

Table. S2. Literature-based analytical verification of the 25 bioactive constituents identified in WN

Category	Target Name	Degree	Betweenness Centrality	Clossness Centrality
Lysosomal Regulation	APOE	41	0.1232	0.6068
Neurodegeneration	APP	38	0.0514	0.5906
Neurodegeneration	RAS	33	0.0546	0.5714
Neuroinflammation	ERK	30	0.0275	0.5432
Neurodegeneration	TP53	30	0.0455	0.5398
Neuroinflammation	BACE1	29	0.0249	0.5333
Neurodegeneration	ULK1	29	0.0303	0.5398
Neurodegeneration	TFEB	29	0.0354	0.5398
Neuroinflammation	RAF	28	0.0273	0.5301
Lysosomal Regulation	SQSTM1	27	0.0689	0.5534
Neurodegeneration	PINK1	27	0.0403	0.5269
Neuroinflammation	MEK	24	0.0329	0.5301
Neurodegeneration	LRRK2	24	0.0201	0.5
Neurodegeneration	TFE3	24	0.0207	0.5207
Neurodegeneration	PSEN1	23	0.021	0.4943
Neurodegeneration	ABCA1	23	0.0285	0.5176
Neurodegeneration	PRKN	23	0.0202	0.5116
Neuroinflammation	PTEN	22	0.0285	0.4888
Neuroinflammation	IL10	20	0.0214	0.4971
Neuroinflammation	TGFB1	20	0.0158	0.4888
Lysosomal Regulation	CTSD	19	0.051	0.4971
Neuroinflammation	TREM2	19	0.0089	0.4808
Neurodegeneration	VCP	19	0.0109	0.5028
Lysosomal Regulation	CTSB	18	0.0185	0.4808
Neurodegeneration	PSEN2	18	0.0098	0.4835

Lysosomal Regulation	LAMP2	17	0.0742	0.5028
Lysosomal Regulation	GRN	16	0.0123	0.4943
Neurodegeneration	FOXO3	16	0.0093	0.4631
Neuroinflammation	NOS3	16	0.049	0.4861

Table S3. Topological analysis of the core target protein in the PPI network

Category	Term	Count	P-Value	Fold Enrichment
BP	Autophagy	12	6.90E-15	40
BP	Astrocyte Activation	16	8.40E-14	15.6
BP	Cellular Response to Amyloid-Beta	7	9.00E-11	89.3
BP	Cholesterol Efflux	12	2.80E-10	15.5
BP	Negative Regulation of Apoptotic Process	7	2.10E-09	56.2
BP	Regulation of Reactive Oxygen Species Metabolic Process	17	2.80E-09	6.8
BP	Positive Regulation of mi-RNA Transcription	7	6.30E-09	47.4
BP	Response to Oxidative Stress	8	1.20E-08	28.4
CC	Lysosomal Lumen	10	2.50E-14	34.6
CC	Neuronal Cell Body	12	4.3E-13	28
CC	Endosome	15	7.5E-10	9.2
CC	Endoplasmic Reticulum	20	1.1E-09	5.7
CC	Membrane Raft	28	2.4E-09	3.7
CC	Extracellular Exosome	12	2.8E-09	12.5
CC	Extracellular Region	31	5.9E-09	3.2

CC	Extracellular Space	34	6.8E-09	2.9
CC	Azurophil Granule Lumen	28	1.2E-08	3.4
CC	Lysosomal Membrane	8	1.3E-07	20.1
CC	Enzyme Binding	13	2.2E-07	7.2
MF	Identical Protein Binding	18	7.4E-12	9.2
MF	Hydrolase Activity	33	9.5E-12	3.9
MF	Protein-Folding Chaperone Binding	28	3.5E-08	3.2
MF	Protein-Containing Complex Binding	7	3.3E-06	17

Table S4. Gene Ontology (GO) enrichment analysis of targets across Biological Process, Cellular Component, and Molecular Function categories.

Term	Count	PValue	Fold Enrichment	Genes
Mitophagy	26	1.71E-13	42	PRKN, APP, VCP, DCTN1, LRRK2, LRP5, PSEN2, PSEN1, PARK7, TNF, MT-ND1, UCHL1, MFN2, NEFL, SPG11, SNCA, PRNP, CYBB, SOD1, FIG4, IL6, PINK1, IL1B, MAPT, TARDBP, SQSTM1
Lysosome	10	2.91E-12	31	PRKN, VCP, PINK1, TFE3, TFEB, MFN2, MITF, FOXO3, TP53, SQSTM1
mTOR signaling pathway	7	7.65E-11	20	GALC, ARSA, SMPD1, HEXB, HEXA, PSAP, GLA
MAPK signaling pathway	9	2.53E-10	17	IL6, TGFB1, PIK3CA, COL4A1, NOS3, IL1B, CYBB, TNF, FOXO1
Pathways of neurodegeneration	16	3.81E-10	28	ARSA, HEXB, GAA, HEXA, MCOLN1, GALC, CLN3, NPC1, NAGLU, NPC2, SMPD1, LAMP2, PSAP, CTSD, GLA, CTSB
Sphingolipid signaling pathway	7	6.70E-04	11	PIK3CA, NOS3, SMPD1, PTEN, TNF, CTSD, TP53
Cholesterol metabolism	5	9.92E-04	14	ABCA1, CYP27A1, NPC1, NPC2, APOE
FoxO signaling pathway	7	0.001052882	10	IL10, IL6, TGFB1, PIK3CA, PTEN, FOXO3, FOXO1
Apoptosis	6	0.006861758	10	PIK3CA, LMNA, TNF, CTSD, TP53, CTSB
Glycosaminoglycan degradation	3	0.011720771	9	NAGLU, HEXB, HEXA
C-type lectin receptor signaling pathway	5	0.013306642	3	IL10, IL6, PIK3CA, IL1B, TNF
PI3K-Akt signaling pathway	9	0.013517329	7	CSF1R, IL6, VWF, PIK3CA, COL4A1, NOS3, PTEN, FOXO3, TP53
Notch signaling pathway	4	0.016819927	10	NOTCH3, NOTCH1, PSEN2, PSEN1
Neurotrophin signaling pathway	5	0.020735942	4	PIK3CA, PSEN2, PSEN1, FOXO3, TP53
NOD-like receptor signaling pathway	6	0.024671198	10	IL6, IL1B, CYBB, MFN2, TNF, CTSB
Inositol phosphate metabolism	4	0.030645424	8	FIG4, SYNJ1, PIK3CA, PTEN
EGFR tyrosine kinase inhibitor resistance	4	0.032690751	9	IL6, PIK3CA, PTEN, FOXO3
Longevity regulating pathway	4	0.043956933	9	PIK3CA, FOXO3, TP53, FOXO1
AGE-RAGE signaling pathway	7	0.047061242	11	CSF1R, TGFB1, IL1B, HSPB1, MAPT, TNF, TP53

Table S5. KEGG pathways associated with shared molecular targets (enrichment profiles retrieved from KEGG database: https://davidbioinformatics.nih.gov/; accessed on 26 March 2026.)

Classification	Molecule Name	ULK1	RAS	RAF	MEK	ERK	JNK	APOE	ABCA1	ABCA7	ABCA4	CYP27A1	DNM2	SORL1	BACE1	MCOLN1	LAMP2	CLN3	NPC1	NPC2	CTSB	CTSD	GAA	SMPD1	GLA	HEXA	HEXB	GALC	ARSA	NAGLU	PSAP	GRN	SQSTM1	TFEB	TFE3	MITF	TERT
Fatty Acids	alpha-Linolenic acid	7.9	9.6	8.9	8.5	7.3	7.3	7	9.3	8.5	8.9	6.9	9.6	9.2	7.4	7.3	7.3	7.7	8.3	8.1	7.6	8.6	7.2	7.6	7.9	8.1	9.1	7.4	8.3	8.5	6.9	8.6	7.3	7	9.6	9.6	9.1
Fatty Acids	Linoleic acid	7.7	7.1	8.8	8.1	7.2	8.2	6.9	9.4	7.6	8.7	7.7	8.3	8.4	7.3	9.6	9	9.5	9.4	8.5	9.5	7.1	7.4	6.9	7.7	7.9	7.6	9.2	7.8	7.6	8.4	7.2	9.1	7	9.7	9	7.4
Fatty Acids	Oleic acid	6.8	9.2	8.8	8.9	9	7	7.8	7.1	9.3	8.6	7.8	7	7.7	7.7	8.9	8.6	9.4	8.2	7.1	8.9	9	8.4	9	8.2	8.3	8	6.9	7.1	6.9	8.6	7.7	8.3	9.4	7.5	8	9
Fatty Acids	Palmitic acid	7.5	7	7.6	7.3	9.5	9.1	8.6	9.3	9.1	7.3	9.4	8.4	9.1	9.4	7.7	7.1	7.5	8	9.2	9.3	6.8	8.3	8	7.4	7.1	7.8	9.5	7.7	8.3	8.8	7.9	9.6	9.6	7.5	8.2	7.7
Fatty Acids	Stearic acid	7.6	6.9	8.6	8.3	6.9	7.6	9.4	7.5	7.2	8.2	9.7	7.5	8.7	9	7.5	8.9	7.9	8.6	8.6	8.4	7.1	9.2	7.7	7.3	6.9	8.5	8.8	6.8	8.3	7.5	8.7	7.3	8.8	7.9	9.5	7.2
Phenolic Acids	Gallic acid	10.2	12.4	12.5	12.7	12.2	10.8	9.5	13.2	10.4	10.2	9.8	9.7	9.4	13.1	11.5	10.7	10.8	8.5	8.4	9.5	7.6	9.1	8.7	8.4	8.1	9.7	9.2	7.8	8.6	8.4	8.6	10.2	13.2	12.9	9.6	8.2
Phenolic Acids	p-Coumaric acid	7.9	9.6	8.9	8.5	7.3	7.3	7	9.3	8.5	8.9	6.9	9.6	9.2	7.4	7.3	7.3	7.7	8.3	8.1	7.6	8.6	7.2	7.6	7.9	8.1	9.1	7.4	8.3	8.5	6.9	8.6	7.3	7	9.6	9.6	9.1
Phenolic Acids	Protocatechuic acid	7.7	7.1	8.8	8.1	7.2	8.2	6.9	9.4	7.6	8.7	7.7	8.3	8.4	7.3	9.6	9	9.5	9.4	8.5	9.5	7.1	7.4	6.9	7.7	7.9	7.6	9.2	7.8	7.6	8.4	7.2	9.1	7	9.7	9	7.4
Phenolic Acids	Sinapic acid	6.8	9.2	8.8	8.9	9	7	7.8	7.1	9.3	8.6	7.8	7	7.7	7.7	8.9	8.6	9.4	8.2	7.1	8.9	9	8.4	9	8.2	8.3	8	6.9	7.1	6.9	8.6	7.7	8.3	9.4	7.5	8	9
Phenolic Acids	Syringaldehyde	7.5	7	7.6	7.3	9.5	9.1	8.6	9.3	9.1	7.3	9.4	8.4	9.1	9.4	7.7	7.1	7.5	8	9.2	9.3	6.8	8.3	8	7.4	7.1	7.8	9.5	7.7	8.3	8.8	7.9	9.6	9.6	7.5	8.2	7.7
Phenolic Acids	Syringic acid	7.6	6.9	8.6	8.3	6.9	7.6	9.4	7.5	7.2	8.2	9.7	7.5	8.7	9	7.5	8.9	7.9	8.6	8.6	8.4	7.1	9.2	7.7	7.3	6.9	8.5	8.8	6.8	8.3	7.5	8.7	7.3	8.8	7.9	9.5	7.2
Tannins	Pedunculagin	10.2	13.2	13.4	12.9	13.6	9.7	9.6	12.4	9.4	9.2	9.7	9.6	10.2	12.2	8.6	8.6	8.4	11.4	9.2	10.7	11.3	10.7	9.9	10.7	8.9	9.6	9.1	8.9	8.7	9.1	8.2	10.6	12.3	12.5	9.3	9.7
Tannins	Tellimagrandin I	10.8	13.5	13.8	12.9	12.8	10.3	7.8	12.6	7.7	7.8	9.4	9.7	9.7	12.1	8.6	8.5	8.3	9.4	8.2	8	8.6	8.4	9.2	6.9	9.5	8.8	9.2	7.4	9.2	6.9	7.9	8.7	12.6	12.1	8.4	9
Tannins	Casuarictin	10.6	12.9	13.1	13.2	13	7.6	8.7	12.3	7.4	7.3	10.7	6.9	6.9	12.2	8	8.3	7.1	6.9	8.1	8.1	7.9	8.3	7.1	6.9	8.9	9	8.6	7.1	8.9	10.7	7.3	9.3	12.3	12	8.3	8
Tannins	Strictinin	11.1	13.4	13.3	12.6	13.2	9.3	8.6	12.6	7.1	6.9	8.9	9.5	8.9	12.6	8.6	8	7.8	8.5	9.7	9.1	7.6	8	7.8	8.5	9.6	9.5	9.4	7.5	7.9	10.8	7.7	9.5	11.8	11.9	9.2	7.7
Flavonoids	Isoquercitrin	10.9	12.1	12.4	11.8	12.2	9.1	7.3	11.7	7.8	8.5	9.6	8.8	9	10.7	8.5	6.9	9.5	8.8	9.2	7.4	9.2	6.9	9.5	8.8	9.1	9.6	9	8	8.2	9.5	9.6	10.5	9.4	9.2	9.1	8.7
Flavonoids	Quercetin	9.7	9.1	10.5	10.1	9.6	8.7	9.2	8.4	8.3	7.5	7.1	9.4	9.4	8.6	7.8	7.8	8.9	9.4	9.4	9.1	8.7	7	7.3	9.4	8.6	6.8	7.1	8.7	6.8	7.3	8.4	8.8	8.7	7.5	8.9	7.5
Triterpenes	Squalene	8.9	9	8.7	9.3	8.7	8.4	7.1	7.9	7.6	7.5	9.6	7.9	9.4	8.6	9.1	8.3	8.5	8.2	7.4	8.9	7.6	6.9	8.7	7.3	9.5	9.6	9.5	7.9	6.8	9.5	8	9.6	9.6	9.3	7.7	7.9
Tocopherols	gamma-Tocopherol	9.3	7.7	7.3	8.4	9.5	8.8	8.5	7.1	8.6	9.7	7.2	8.3	9.3	8.9	8.8	8.8	7.8	7.7	9.1	9.1	9.3	9.4	8.3	8.3	9.1	8.7	8.8	9.1	9.4	7.8	7.9	7.1	8.5	6.9	8.2	8.4
Tocopherols	delta-Tocopherol	7.6	8.5	6.9	6.9	9.2	7.8	7.2	8.3	9	7.4	8.6	7	6.9	8.3	8.4	8.6	8.9	9.3	8.3	7.7	9.1	7.6	8.1	7	6.9	9.6	9.2	8.8	8	7.3	7.3	7.5	8.4	8.9	8.7	7.6
Amino Acids	L-Arginine	9.3	12.4	12.5	11.9	12.1	9.4	9.7	11.5	8.2	9.2	7.8	7.3	6.9	12.2	8.9	9.3	8.4	9.5	7.6	9.5	8.9	9.3	8.4	10.2	8	7.5	8.4	6.9	8.4	8.6	8.8	10.3	12.4	12.2	7.2	8.2
Amino Acids	Tryptophan	6.8	9.2	8.8	8.9	9	7	7.8	7.1	9.3	8.6	7.8	7	7.7	7.7	8.9	8.6	9.4	8.2	7.1	8.9	9	8.4	9	8.2	8.3	8	6.9	7.1	6.9	8.6	7.7	8.3	9.4	7.5	8	9
Amino Acids	Spermidine	9.4	12.4	12.6	11.8	11.4	8.6	9	11.7	8.6	7.1	8.9	11.9	9.4	11.6	7.9	7.7	8.2	7.4	7.3	10.7	6.9	6.9	8.5	8.8	9.4	7.3	8.3	7	10.2	7.3	9.5	10.4	12	11.6	8.3	9.2
Phospholipids	Phosphatidylcholine	9.1	9.4	7.7	7.1	7.5	8	9.2	9.3	6.8	8.3	8	7.4	7.1	7.8	9.5	7.7	8.3	8.8	7.9	9.6	9.6	7.5	8.2	7.7	8.9	9.5	8.9	9.6	9.1	8.6	9.5	9.1	6.9	9.7	7.5	7.5
Phospholipids	Phosphatidylethanolamine	8.7	9	7.5	8.9	7.9	8.6	8.6	8.4	7.1	9.2	7.7	7.3	6.9	8.5	8.8	6.8	8.3	7.5	8.7	7.3	8.8	7.9	9.5	7.2	9.6	8.8	9	9.5	9.6	6.8	9.6	8.7	9.6	9	8	8.2

Positive-control Ligand	Sotorasib	9.6	14.4	14.1	13.9	13.8	10.8	8.1	9.1	8.4	9.1	8.9	9.1	7.7	9.5	8.3	8.7	9.2	8.9	7.9	8.2	6.8	6.8	7.1	6.9	8.9	9.5	8.9	9.6	9.1	8.6	9.5	8.3	9.5	7.8	8.1	8.6
Positive-control Ligand	Vemurafenib	8.9	14.1	14.2	13.9	13.2	11.1	8.9	8.5	7.8	8.9	8.4	9.4	7.5	8.2	8.7	7.9	9.1	8.8	8.1	7.9	8.3	7.9	7.8	9.5	8.9	8.5	7.8	7.8	8.3	8.8	8.6	9.3	8.6	7.7	9.6	7.8
Positive-control Ligand	Trametinib	9.4	13.6	14	14.6	14.3	11.8	9.4	8.9	8.3	7.3	7.3	7	9.2	9.4	7.4	9.1	8.3	7.6	7.1	7.3	7.4	7.1	7.5	8	8.7	7.9	9.1	8.8	9.5	7.3	8.7	8.3	8.1	8.4	8.8	9.5
Positive-control Ligand	Ulixertinib	8.6	13.7	14.2	14.5	14.7	12.1	7.8	7.3	6.9	8.5	7.5	7.8	9.5	7.3	8.7	8.3	8.1	8.4	9	9.5	8.9	9.6	9.1	8.6	9.5	9.1	6.9	8.9	8.9	8.7	9.5	7.7	7.4	9.2	9	7.3
Positive-control Ligand	GW3965	9.8	8.3	8.2	8	9.1	7.6	9.2	14.3	13.7	14.4	9.3	6.9	8.3	9.7	9.7	8.8	7.8	8.9	7.5	7.9	7.3	8.3	7	8.4	7.7	9.1	8.7	6.9	6.9	7.1	6.9	8.6	7.7	7.2	8.2	9.1
Positive-control Ligand	Curcumin	9.4	7.7	8.9	9.6	9.3	9.5	9.8	7.5	9.3	8.9	9.5	8.9	9.3	8.6	8.8	8.4	9.5	9.3	9.4	9.3	8.4	10.2	9.5	7.9	9.5	8.9	9.3	8.4	10.2	10.2	9.1	10.6	11.1	11.3	10.5	8.6

Table S6. Predicted binding affinities of walnut bioactive constituents and positive-control ligands toward the core therapeutic targets. (- kcal/mol)
